# Identification of novel aphid‐killing bacteria to protect plants

**DOI:** 10.1111/1751-7915.13902

**Published:** 2021-08-01

**Authors:** Deepa Paliwal, Amanda J. Hamilton, Glyn A. Barrett, Fabrizio Alberti, Helmut van Emden, Caroline L. Monteil, Tim H. Mauchline, Ralf Nauen, Carol Wagstaff, Chris Bass, Robert W. Jackson

**Affiliations:** ^1^ School of Biological Sciences University of Reading Whiteknights Reading RG6 6AH UK; ^2^ School of Life Sciences The University of Warwick Coventry CV4 7AL UK; ^3^ Aix‐Marseille Université CEA CNRS BIAM Saint Paul lez Durance 13108 France; ^4^ Rothamsted Research Harpenden Herts AL5 2JQ UK; ^5^ Crop Science Division Bayer AG Monheim 40789 Germany; ^6^ School of Chemistry, Food and Pharmacy University of Reading Whiteknights Reading RG6 6AH UK; ^7^ University of Exeter Penryn Cornwall TR10 9FE UK; ^8^ School of Biosciences and Birmingham Institute of Forest Research University of Birmingham Edgbaston Birmingham B15 2TT UK

## Abstract

Aphids, including the peach‐potato aphid, *Myzus persicae,* are major insect pests of agriculture and horticulture, and aphid control measures are limited. There is therefore an urgent need to develop alternative and more sustainable means of control. Recent studies have shown that environmental microbes have varying abilities to kill insects. We screened a range of environmental bacteria isolates for their abilities to kill target aphid species. Tests demonstrated the killing aptitude of these bacteria against six aphid genera (including *Myzus persicae*). No single bacterial strain was identified that was consistently toxic to insecticide‐resistant aphid clones than susceptible clones, suggesting resistance to chemicals is not strongly correlated with bacterial challenge. *Pseudomonas fluorescens* PpR24 proved the most toxic to almost all aphid clones whilst exhibiting the ability to survive for over three weeks on three plant species at populations of 5–6 log CFU cm^−2^ leaf. Application of PpR24 to plants immediately prior to introducing aphids onto the plants led to a 68%, 57% and 69% reduction in aphid populations, after 21 days, on *Capsicum annuum*, *Arabidopsis thaliana* and *Beta vulgaris* respectively. Together, these findings provide new insights into aphid susceptibility to bacterial infection with the aim of utilizing bacteria as effective biocontrol agents.

## Graphical abstract

An aphid‐feeding system was adapted to screen environmental bacteria for aphid‐killing properties and a selection of bacteria exhibited low‐to‐high killing effects in a range of aphid species including insecticide‐resistant clones. *Pseudomonas fluorescens* PpR24 demonstrated the best killing effect *in vitro*, whilst being able to survive on plant leaves over three weeks. Application of PpR24 to plants immediately prior to introducing aphids onto the plants led to a 50–60% reduction in aphid populations, after 21 days, on three different plant species.

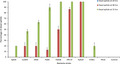

## Introduction

There are more than 5000 described species of aphids (Hemiptera: Aphididae), of which around 100 are considered major insect pests of agriculture and horticulture (Blackman and Eastop, 2000). These species cause damage to many economically important crop plants through direct feeding and/or as efficient vectors of numerous plant viruses. Current aphid control measures rely heavily on the use of insecticides such as carbamates, pyrethroids, neonicotinoids, tetramic acids, and chordotonal organ modulators such as flonicamid/pymetrozine (Bahlai *et al*., 2010; Bass *et al*., 2014). The active target for many of these chemicals is the insect central nervous system, leading to disruption of nerve impulse transmission and death. Insect populations, however, can rapidly evolve resistance to insecticides, thus rendering these chemicals ineffective and hampering long‐term control.

The peach‐potato aphid, *Myzus persicae*, is recognized as one of the most important agricultural pests worldwide. This is in part due to its wide host range and ability to feed on more than 400 species of plants across 40 different families (Blackman and Eastop, 2000; van Emden and Harrington, 2007). *M. persicae* has proved to be exceptionally prone at evolving resistance to the insecticides used for control, leading to widespread and multiple resistance in global populations (Bass *et al*., 2014). Several genetically independent mechanisms of resistance have been described (Bass *et al*., 2014) including: (i) metabolic resistance involving the increased production of detoxifying enzymes (esterases and P450s) that metabolize or sequester the insecticide before it reaches its target protein. This form of resistance has been primarily demonstrated for organophosphates and neonicotinoids, although carbamates and pyrethroids are also known to be affected to a lesser extent; (ii) target‐site resistance mechanisms, which involve structural alteration of the insecticide target protein that renders it less sensitive to the toxic effect of the insecticide. These alterations are generally driven through specific mutation of genes encoding acetylcholinesterase, the voltage‐gated sodium channel and the nicotinic acetylcholine receptor, which in turn confer high levels of resistance to pirimicarb, pyrethroids and neonicotinoids respectively; and (iii) reduced penetration of insecticide through the cuticle, primarily through cuticle thickening and composition modification.

The development of insecticide resistance in *M. persicae* represents a serious threat to the sustainable control of this species and alternative means of control are urgently required to support integrated pest management (IPM) strategies. Some of the most promising methods, under current development, include biopesticides or compounds derived from or produced by living organisms. Microbial derived biopesticides include specialist bacterial or fungal entomopathogens that may be delivered as whole organisms or as cocktails of purified metabolites in formulation (Haas and Keel, 2003; Haas and Defago, 2005; Jousset *et al*., 2011; Mendes *et al*., 2011). For example, Bt (*Bacillus thuringiensis* formulated as a biopesticide) is an important biopesticide for controlling several pest species, and in 2011 accounted for around 1% of the total market of insecticides (Sparks and Nauen, 2015). Bacterial species residing in and recovered from disease‐suppressive soils as well as the plant phylloplane and rhizosphere are strong candidates for use as novel biocontrol agents. Direct antagonism by indigenous phylloplane bacteria has been shown to be useful as biocontrol strategies in controlling populations of pathogens (Halfeld‐Vieira *et al*., 2015). From this perspective, native phylloplane microorganisms, with intrinsic abilities to acquire nutrients from their environment and grow and maintain populations, are good candidates for biocontrol (Wilson and Lindow, 1994a,b; Mercier and Lindow, 2000; Smith and Lindow, 2013). These microbes may suppress or eliminate pest populations through the secretion of toxins and other secondary metabolites by the antagonist. In addition, some plant‐associated bacteria have the ability to trigger induced systemic plant resistance (ISR), thus preconditioning plant defences prior to infection by a pathogen (Halfeld‐Vieira *et al*., 2006; Romeiro *et al*., 2010).

Several soil‐ and plant‐associated bacteria including plant pathogens and beneficial bacteria (*B. thuringiensis*, *Dickeya dadantii*, *Pseudomonas syringae*, *P*. *protegens*, *P*. *chlororaphis*) have the ability to kill insects in orders Hemiptera, Diptera, Coleoptera and Lepidoptera (Grenier *et al*., 2006; Péchy‐Tarr *et al*., 2008; Costechareyre *et al*., 2012; Smee *et al*., 2017; Hendry *et al*., 2018; Vesga *et al*., 2020; Smee *et al*., 2021). The mechanisms underpinning this process have been studied in *B*. *thuringiensis* through work on Cry toxins against Lepidoptera, but novel systems can also target aphids, for example Bt#BREF24 isolate secretes the binary toxin, Vip2Ae‐Vip1Ae and novel Cry proteins Cry41Ab1 and Cry41Aa1 from Bt strain H1.5 (Sattar and Maiti, 2011; Palma *et al*., 2014a). These observations point to a more intimate relationship between insects and bacteria than previously realized. Indeed, we can posit that plant‐dwelling bacteria ingested by plant‐feeding insects have evolved adaptations to cope with insect ingestion or perhaps even to exploit insects as a dispersal mechanism (Dorati *et al*., 2018; Flury *et al*., 2019; Vesga *et al*., 2020).

Based on these properties, we sought to examine plant‐ and soil‐based bacteria to identify those that kill aphids and to measure the efficacy of killing. We also aimed to test whether these bacteria could kill insecticide‐resistant aphids and whether the bacteria could be used to reduce aphid populations on plants. Together, our study shows that a wide range of bacteria have the ability to kill aphid pests, including insecticide‐resistant clones, and that bacterial application to plants could reduce aphid colonization, thus indicating potential use in biocontrol strategies.

## Results

### Isolation and identification of aphid‐killing bacteria

**Fig. 1:**
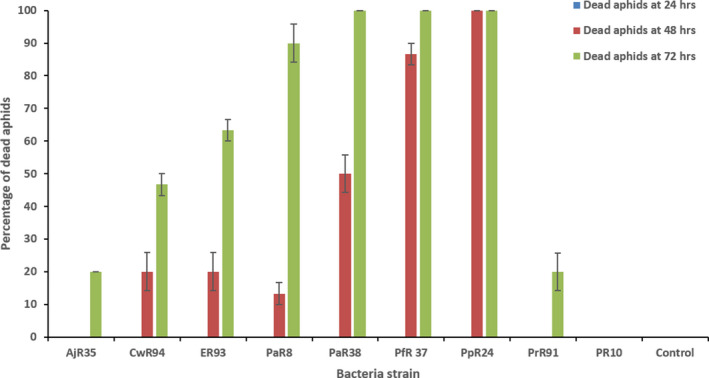
Assessment of aphid mortality by various plant‐associated bacteria strains. Mortality assay showing the percentage of dead *Myzus persicae* clone 4106A (*N* = 10) at 72 h after ingestion of artificial diet inoculated with various bacterial cells (10^7^ CFU ml^−1^). Bacterial strain tested – *Acinetobacter* sp. AjR35, *Enterobacte*r sp. CwR94, *Enterobacter* sp. ER93, *Pantoea* sp. PaR8, *Pantoea agglomerans* PaR38, *Pseudomonas fluorescens* PfR37, *P. fluorescens* PpR24, *Pseudomonas rhizosphaerae* PrR91 and *Pseudomonas* sp. PR10. Error bars represent standard error of the mean of three biological replicates.

Ten different plant species, a lake water sample and an invertebrate identified as *Broscus cephalotes*, were sampled (Table S1) and homogenized to isolate and purify bacteria on KB, LB and M9. In total, 140 bacterial strains were isolated and used in initial aphid *in vitro* screening tests (ten aphids challenged via oral feeding assay) to assess toxicity. Of these 140 strains, nine, originating from a range of different plants and locations (Table S1), showed toxicity towards *M. persicae* (Fig. 1). Around 10–100% of aphids died at 48 h after feeding on six strains, CwR94, ER93, PaR8, PaR38, PfR37 and PpR24. After 72 h, all strains revealed variation in the efficiency of their aphid‐killing ability. The maximum mortality (90–100%) was caused by four strains PaR8, PaR38, Pfr37 and PpR24 at 72 h suggesting these are the most effective aphid‐killing strains. Culture filtrates from these four strains were tested for the ability to kill aphids, but no aphid death was observed (data not shown) suggesting the action of killing was not solely due to a secreted product and required live cells. Putative identifications via 16S rRNA sequence analysis revealed that four bacteria (including PpR24) were Pseudomonads, four were Enterobacteriaceae closely related to *Enterobacter* and *Pantoea* and a single species belonged to the *Acinetobacte*r group (Table 1). A phylogenetic analysis of the *P. fluorescens* species complex revealed the presence of two clades with at least five subgroups with strains previously classified as *P. fluorescens*, interspersed with strains classified in other species (Fig. S1). PpR24 was observed to reside in subclade 1 and to be closely related to *P*. *fluorescens* SS101, which was isolated from wheat roots in the Netherlands (Fig. S1). The subclade 1 also includes previously sequenced *P*. *fluorescen*s strains SBW25, A506, NZ052, PCL1571 and EGD‐AQ6.

**Table 1:** 16S rRNA sequence analysis of new aphid‐killing bacteria (highest similarity match using the BLAST database).

Strain	Source	Homologous microorganism (% identity)
PaR8	Isolated from leaf of *Capsicum annuum*, Private garden, Reading	*Pantoea* sp. (97%)
PR10	Isolated from leaf of *Solanum lycopersicum*, Private garden, Reading.	*Pseudomonas* sp. G1329 (98%)
ER93	Isolated from leaf of *Capsicum annuum*, Cantelo Nursery, Reading	*Enterobacter xiangfangensis* strain ADA‐20 16S (98%)
PpR24	Isolated from root of *Brassica oleracea*, Experimental greenhouse, University of Reading	*Pseudomonas poae* strain UASWS1796 (99%)
AjR35	Isolated from leaf of *Hamamelidae fagale*, Harris garden, University of Reading	*Acinetobacter* sp. strain XS (99%)
CwR94	Isolated from leaf of *Fragaria ananassa*, Experimental greenhouse, University of Reading	*Enterobacter* sp. strain LA12P41 (98%)
PrR91	Isolated from leaf of *Foeniculum vulgare*, Private garden	*Pseudomonas rhizosphaerae* GAPP71 (99%)
PaR38	Isolated from leaf of *Nasturtium officinale*, Experimental greenhouse, University of Reading	*Pantoea agglomerans* mL16 (99%)
PfR37	Isolated from leaf of *Calendula officinalis*, Harris garden, University of Reading	*Pseudomonas fluorescens* strain BTGOIC‐10 (99%)

### Aphid toxicity tests

With initial tests revealing the pathogenic potential of nine bacterial isolates against *M. persicae,* we aimed to determine the effect of these pathogens on other aphid species. Toxicity bioassays revealed the killing effect was also observed on five other aphid species, *Aphis fabae*, *Brevicoryne brassicae, Macrosiphum albifrons, Nasonovia ribsnigri* and *Aulacorthum solani* (Fig. S2A–E, Table S2). Variation in sensitivity of these species to the nine bacterial species were observed, for example *B. brassicae* appeared to be particularly susceptible to all the bacteria tested, exhibiting rapid mortality in the first 24 h following bacterial ingestion. Conversely, *M. albifrons* appeared to be more resistant. Of the bacterial strains tested, *Pseudomonas fluorescens* PpR24 displayed the greatest efficacy against the most aphid species.

### Relative sensitivity of insecticide‐resistant (IR) *M. persicae* clones to bacterial exposure

**Fig. 2:**
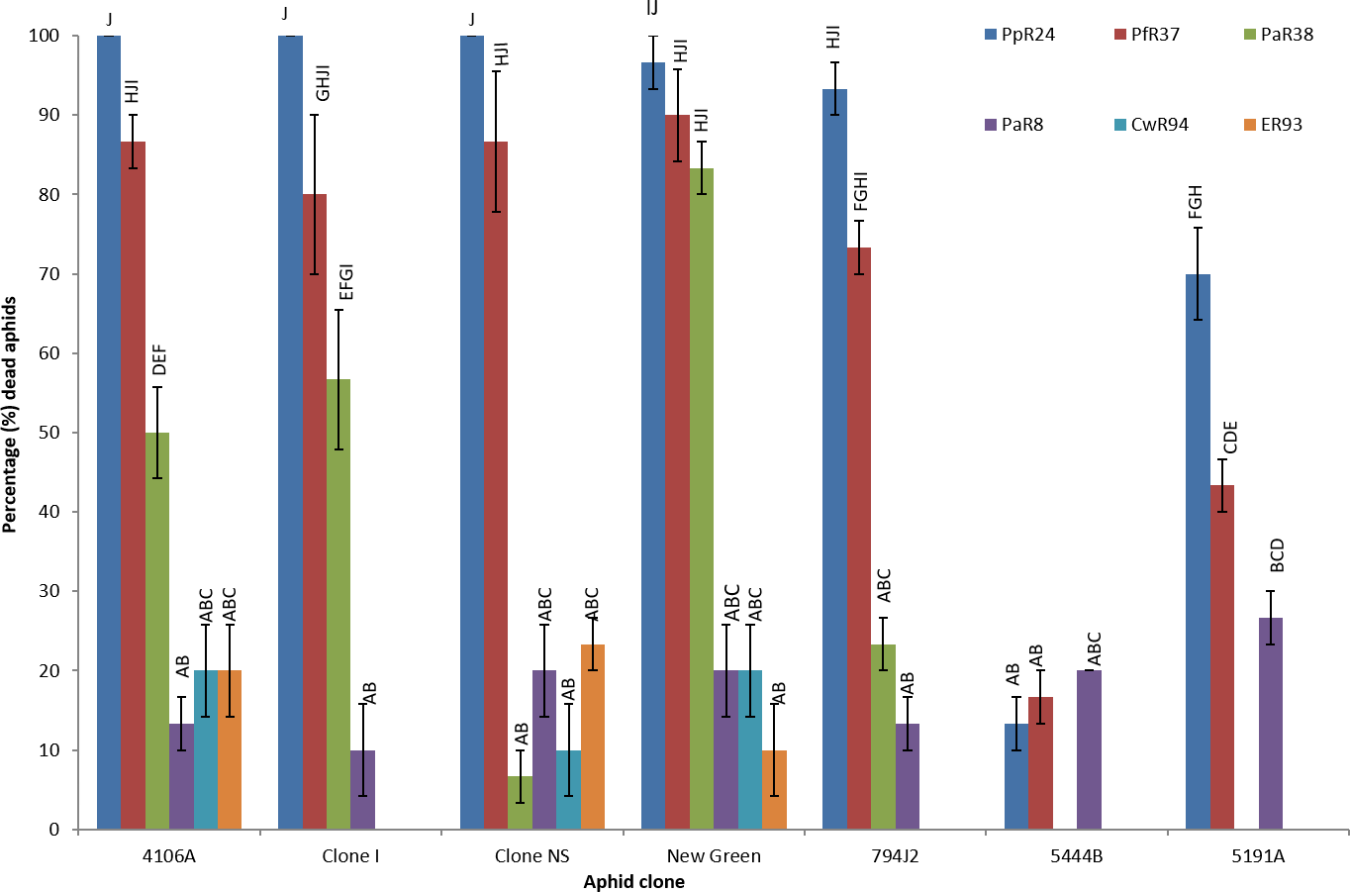
Assessment of aphid mortality caused by selected bacteria. Mortality assay showing the percentage of dead aphids (*N* = 10) at 48 h after ingestion of artificial diet inoculated with cells of various bacterial species (10^7^ CFU ml^−1^). Error bars represent standard error of the mean of three biological replicates. ANOVA detected statistically significant differences (*P* < 0.05) and comparison of means by Tukey–Kramer HSD were shown as letters (where different letters on the graphs indicate statistically significant differences). Aphid clones – three susceptible clones ‘4106A‐SUS 1’, ‘4225B‐SUS 2’ and ‘Clone‐NS SUS 3’ and four resistant clones ‘New green – RES 1’, ‘794J2 – RES 2’, ‘5191A – RES 3’ and ‘5444B – RES 4’. Bacterial strains tested – *Pseudomonas fluorescens* PfR37, *P. fluorescens* PpR24, *Pantoea* sp. PaR8, *Pantoea agglomerans* PaR38, *Enterobacte*r sp. CwR94 and *Enterobacter* sp. ER93.

Based on the screening of the bacterial pathogens against the different aphid species, we were able to categorize their efficacy as low (30–50%), moderate (50–80%) or high (90–100%) based on percentage of aphid mortality. Variations in aphid susceptibility and resistance to chemical pesticides allow for a similar qualitative classification. We therefore sought to investigate whether the variations seen for chemical resistance and aphid mortality were correlated. For example, it is feasible that IR and insecticide‐susceptible (IS) clones of the same species will show differences in susceptibility to the bacterial pathogens that might, in turn, elaborate upon the mechanisms of toxicity. To determine this, a collection of *M. persicae* clones with variable IR mechanisms were screened for their susceptibility to bacterial challenge compared with insecticidal susceptible (IS) clones. The preliminary screening found that six bacterial strains (PpR24, PaR38, CwR94, PaR8, PfR37 and ER93) could be classified as 50–100% pathogenic to all tested aphid clones at 72h while the other three strains (AjR35, PrR91 and PR10) were categorized as ‘low’ and ‘non‐toxic’ to all tested aphid clones (Fig. 2; Fig. S3). These six highly virulent aphid‐killing bacteria were selected for subsequent trials and further analysis enabling us to determine which aphid clones were more or less fit to bacterial challenge using different inoculation doses (10^2^–10^7^ CFU ml^−1^) and time points (48 and 72 h).

*Pseudomonas fluorescens* PpR24 and PfR37 led to the highest mortality (90–100%) to all UK‐ IR and IS clones after 48 h. For *Pantoea agglomerans* PaR38, 20–80% mortality was observed in all UK‐ IR and IS clones whereas a lower mortality of 20–40% was associated with *Pantoea* sp. PaR8, and both *Enterobacter* strains (CwR94 and ER93) (Fig. S4A–C). At higher infective doses (10^7^ CFU ml^−1^), two clones from mainland Europe, 5191A and 5444B, were found to be less sensitive to *P. fluorescens* PpR24 and PfR37 and *Pantoea* sp. PaR8 with a 20‐70% mortality (Fig. S4D and E). For all bacteria, lower infective doses of 10^5^–10^6^ CFU ml^−1^ resulted in a 20–100% death in all UK‐IR aphid clones with no deaths whatsoever for the European 5191A and 5444B aphid clones. Concentrations below 10^5^ CFU ml^−1^ resulted in no mortality across all trials (all combinations of bacteria and aphids) (Fig. S4A–G). No aphid mortality was recorded in control sachets (Mittler diet without bacteria). Aphid mortality on higher bacterial concentration (10^6^–10^7^ CFU ml^−1^) sachets showed highly significant differences amongst the treatments. Conversely, lower concentrations ranging between 10^2^ and 10^5^ CFU ml^−1^ showed similar mortality rate in all aphid clones with no significant differences.

After 72 h, six strains *P. fluorescens* PpR24 and PfR37, *Pa. agglomerans* PaR38, *Pantoea* sp. *PaR8*, and both *Enterobacter strains* (CwR94 and ER93), had resulted in 80–100% aphid mortality. They were toxic to all three IS aphids (4106A, Clone‐clone‐NS and 4225B) and two UK‐IR aphids at bacterial cell concentrations ranging from 10^5^ to 10^7^ CFU ml^−1^. However, at lower bacterial concentrations a reduced mortality of 20–50% mortality was observed (Fig. S5A,B,C,F and G). Dose‐dependent mortality was similarly observed for the two *Enterobacter* strains with 60–80% effectiveness in all UK‐IR and IS aphids at 10^7^ CFU ml^−1^, whereas at lower concentrations mortality was reduced to 20–50% (Fig. S5A,B,C,F andG). For 5191A (RES 3) and 5444B (RES 4), only three strains, *P. fluorescens* PpR24 and PfR37 and *Pantoea* sp. PaR8, caused 40–100% mortality at 10^6^–10^7^ CFU ml^−1^ whereas lower concentrations caused only 20–30% mortality (Fig. S5D and E). *Pa. agglomerans* was considered as moderately pathogenic to 5191A (RES 3) and 5444B (RES 4) and caused 70% and 50% mortality, respectively, with a lowered total mortality of 10–20% mortality for two associated strains (Fig. S5D and E). There was a statistically significant difference between the bacterial treatments mainly observed at lower concentrations ranging between 10^2^ and 10^5^ CFU ml^−1^ which were shown by different letters.

To assess generalized pathogenicity of various bacteria on IR and IS aphids, analysis of variance compares the variability in mortality readings (at 72 h) of all aphid clones for each bacterial treatment with bacterial strains, aphid clones and infection doses as test parameters (Table S3). The ANOVA results suggested that the means mortality strongly varies with all parameters. The presence of significantly (*P* < 0.001) strong interactions between all parameters explained substantial variability in the aphid mortality (Table S3).

To establish the relative efficacy of aphid killing, the mean lethal concentration of 50 (LC_50_ – the concentration which kills 50% of the test population) was calculated for each aphid clone. This allows a comparison of the susceptibility of clones and ability to estimate a ‘Tolerance factor’, which is the ratio between the LC_50_ values of the IR/IS clone with the laboratory IS clone.

The tolerance factor (TF) of the New green (RES 1) aphid for all six pathogenic bacteria was lower than 1.00 (Table 2), indicating greater susceptibility to bacterial challenge than its reference IS clone 4106A. Conversely, UK‐IR clone 794J2 (RES 2) showed variance in susceptibility towards different bacteria. Clone 794J2 (RES 2) had a lower TF (< 1.00) for *P. fluorescens* PpR24 and both *Enterobacter* strains, whereas it was slightly resistant (1.8–2.5‐fold increase) to *P. fluorescens* PfR37 and *Pa. agglomerans* compared with reference IS clone 4106A. The results showed no statistical significance (*P* < 0.05) in LC_50_ values due to overlapping upper and lower doses for each of the UK ‐IS and IR clones in all bacterial treatments. 5444B was the most resistant to all bacterial species except for *Pantoea* sp. PaR8 where it was more sensitive than 4106A with a reduced TF of 0.49. 5191A was also more sensitive to both *P. fluorescens* PpR24 and *P*. *fluorescens* PfR37 than the reference IS clone 4106A with a significant reduction in TF to 0.22 and 0.47 respectively. For the remaining four bacterial strains, 5444B was identified as more resistant having a greater TF (Table 2). There was statistical significance in LC_50_ values between the UK ‐IS and two Europe‐IR clones in all bacterial treatments.

**Table 2:** Feeding bioassay (sachets) results with different bacteria against insecticide‐susceptible and insecticide‐resistant aphid clones.

Aphid	Bacteria	Bioassay location & physical conditions	*P. fluorescens* PpR24	*P. fluorescens* PfR37	*Pantoea* sp. PaR8	*Enterobacter* sp. CwR94	*Enterobacter* sp. ER93	*Pa. agglomerans* PaR38
4106A (SUS 1)	LC_50_ (Bacterial CFU ml^−1^)	Set I Aphid rearing room (University of Reading) at 21°C, 16‐h light/8‐h dark) regime	5.22 × 10^2^	4.87 × 10^4^	1.16 × 10^4^	1.12 × 10^7^	6.53 × 10^6^	1.37 × 10^4^
	95% confidence limits		3.5 × 10^2^ – 7.55 × 10^2^	3.13 × 10^4^ – 7.45 × 10^4^	3.22 × 10^3^ – 3.68 × 10^4^	9.57 × 10^6^ – 1.34 × 10^7^	5.44 × 10^6^– 7.97 × 10^6^	5.38 × 10^3^ – 3.375 × 10^4^
New green (RES 1)	LC_50_ (Bacterial CFU ml^−1^)		1.55 × 10^2^	2.89 × 10^4^	1.22 × 10^3^	2.2 × 10^6^	2.89 × 10^6^	9.4 × 10^3^
	95% confidence limits		7.7 × 10^1^ – 2.62 × 10^2^	2.31 × 10^4^ – 3.62 × 10^4^	1.32 × 10^2^ – 5.1 × 10^3^	1.59 × 10^6^ – 3.13 × 10^6^	2.12 × 10^6^ – 4.07 × 10^6^	6.19 × 10^3^ – 1.43 × 10^4^
	Tolerance Factor^a^		0.3	0.59	0.1	0.2	0.44	0.69
794J2 (RES 2)	LC_50_ (Bacterial CFU ml^−1^)		3.99 × 10^2^	8.77 × 10^4^	1.37 × 10^4^	2.47 × 10^6^	1.11 × 10^6^	3.43 × 10^4^
	95% confidence limits		2.38 × 10^3^ – 5.23 × 10^3^	1.63 × 10^4^ – 4.98 × 10^4^	2.47 × 10^3^ – 1.59 × 10^4^	1.97 × 10^4^– 7.28 × 10^4^	3.54 × 10^3^– 2.44 × 10^4^	3.17 × 10^3^– 1.57 × 10^4^
	Tolerance Factor^a^		0.77	1.8	1.18	0.22	0.17	2.51
4106A (SUS 1)	LC_50_ (Bacterial CFU ml^−1^)	Set II Specialist containment Insectary (Rothamsted research) at 21°C, 16‐h light/8‐h dark) regime	9.28 × 10^3^	1.42 × 10^5^	2.15 × 10^5^	9.43 × 10^6^	1.57 × 10^7^	4.15 × 10^5^
	95% confidence limits		6.34 × 10^3^ – 1.36 × 10^4^	9.81 × 10^4^ – 2.06 × 10^5^	1.34 × 10^5^ – 3.45 × 10^5^	7.19 × 10^6^ – 1.32 × 10^7^	1.04 × 10^7^ – 2.79 × 10^7^	2.67 × 10^5^ – 6.6 × 10^5^
5191A (RES 3)	LC_50_ (Bacterial CFU ml^−1^)		2.08 × 10^3^	6.73 × 10^4^	4.81 × 10^5^	6.38 × 10^7^	6.38 × 10^7^	2.80 × 10^6^
	95% confidence limits		1.09 × 10^3^ – 3.85 × 10^3^	3.60 × 10^4^ – 1.27 × 10^5^	2.82 × 10^5^ – 8.63 × 10^5^	3.40 × 10^7^ – 1.62 × 10^8^	3.40 × 10^7^ – 1.62 × 10^8^	1.20 × 10^6^ – 8.76 × 10^6^
	Tolerance Factor^a^		0.22	0.47	2.24	6.77	4.07	6.75
5444B (RES 4)	LC_50_ (Bacterial CFU ml^−1^)		4.95 × 10^4^	6.51 × 10^5^	1.07 × 10^5^	4.17 × 10^7^	8.65 × 10^7^	4.42 × 10^6^
	95% confidence limits		3.39 × 10^4^ – 7.23 × 10^4^	4.81 × 10^5^ – 8.97 × 10^5^	7.12 × 10^4^ – 1.60 × 10^5^	2.26 × 10^7^ – 1.30 × 10^8^	3.77 × 10^7^ – 3.73 × 10^8^	1.97 × 10^6^ – 1.28 × 10^7^
	Tolerance Factor^a^		5.33	4.58	0.5	4.42	5.51	10.64
4106A (SUS 1)	LC_50_ (Bacterial CFU ml^−1^)	Set III Controlled growth cabinet (University of Reading) at 21°C, 16‐h light/8‐h dark) regime	1.1 × 10^2^	1.63 × 10^4^	6.24 × 10^4^	1.48 × 10^7^	2.92 × 10^6^	3.71 × 10^5^
	95% confidence limits		5.8 × 10^1^ – 1.92 × 10^2^	1.08 × 10^4^ – 2.45 × 10^4^	2.72 × 10^4^ – 1.47 × 10^5^	9.27 × 10^6^ – 2.77 × 10^7^	1.88 × 10^6^ – 4.91 × 10^6^	2.04 × 10^5^ – 7.14 × 10^5^
4225B (SUS 2)	LC_50_ (Bacterial CFU ml^−1^)		3.3 × 10^2^	6.53 × 10^4^	3.53 × 10^4^	1.10 × 10^7^	9.04 × 10^6^	2.19 × 10^4^
	95% confidence limits		1.39 × 10^2^ – 6.49 × 10^2^	6.48 × 10^4^ – 6.58 × 10^4^	1.64 × 10^4^ – 7.50 × 10^4^	7.56 × 10^6^ – 1.81 × 10^7^	6.45 × 10^6^ – 1.42 × 10^7^	7.15 × 10^3^ – 6.57 × 10^4^
	Tolerance Factor^a^		0.64	1.34	3.02	0.98	1.38	1.6
Clone‐NS (SUS 3)	LC_50_ (Bacterial CFU ml^−1^)		6.9 × 10^1^	3.13 × 10^4^	1.79 × 10^4^	3.49 × 10^4^	2.47 × 10^6^	6.68 × 10^4^
	95% confidence limits		5.24 × 10^1^ – 9.00 × 10^1^	1.56 × 10^4^ – 6.30 × 10^4^	6.51 × 10^3^ – 4.68 × 10^4^	2.08 × 10^6^ – 6.64 × 10^6^	1.49 × 10^6^ – 4.56 × 10^6^	3.17 × 10^4^ – 1.41 × 10^5^
	Tolerance Factor^a^		0.63	1.92	0.29	0.24	0.85	0.18

To calculate LC_50_ values of each bacterium for all aphid clones, 72 h aphid mortality readings at six bacterial concentrations ranging from 10^7^ to 10^2^ CFU ml^‐1^ were considered. The LC_50_ dose of each bacterium for each aphid clone is shown along with lower and upper concentrations values at 95% confidence limits. The calculated Tolerance Factor for the four resistant and susceptible aphid clones is also shown. A reference 4106A (IS) aphid clone was used for calculating the resistance ratio at different physical laboratory conditions.

^a^ Tolerance Factor is a ratio of the LC_50_ value of the tested resistant/susceptible clone to the LC_50_ value of the laboratory susceptible clone.

To strengthen any correlation between bacterial and insecticidal susceptibility, two more IS reference clones (4225B and Clone‐NS; UK and Europe, respectively) were tested. 4225B showed greater susceptibility (TF = 0.64) than 4106A to *P. fluorescens* PpR24. 4225B was more resistant to *Pantoea* sp. PaR8 challenge than 4106A (TF = 3). Interestingly, similar TF values for other bacterial species, upon comparison, to 4106A were observed (Table 2) with no significant variation among the treatments.

The TF of Clone‐NS for all pathogenic bacteria was lower than 1, indicating greater susceptibility towards bacterial challenge compared with its reference IS clone 4106A, with an exception of slight resistance to *P. fluorescens* PfR37 (TF = 1.92; Table 2) but were not significantly different from each other.

### Bacterial quantification in infected aphids

**Fig. 3:**
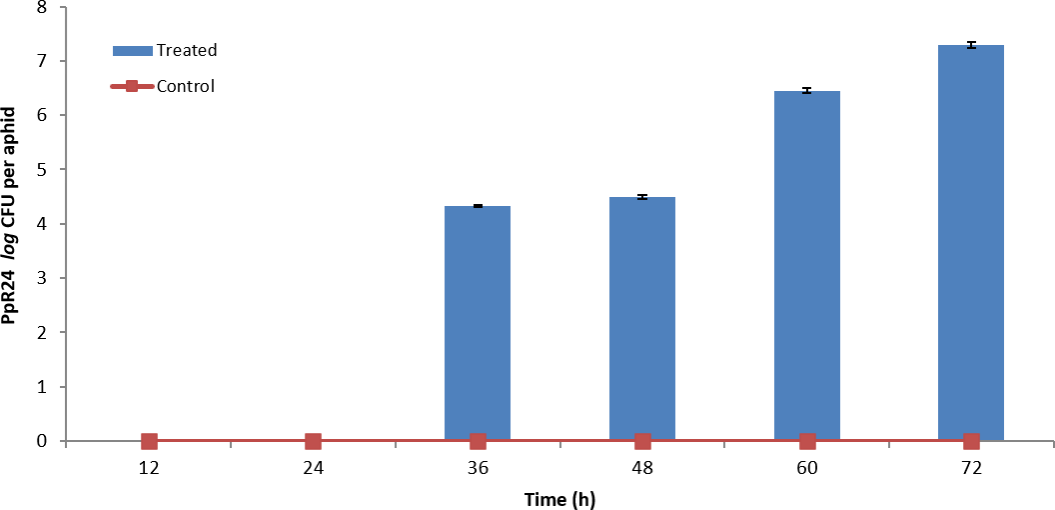
*Pseudomonas fluorescens* PpR24 population growth inside aphid clone 4106A. *P. fluorescens* PpR24 populations within infected 4106A aphids were continually elevated to 2 × 10^7^ CFU/aphid over the period of inoculation and no colonies were recovered from control aphids for the entire duration of the experiment. Control: Ten aphids were fed in sterile diet with three replicates (*N* = 3). Treated: Ten aphids, infected with 10^2^ CFU ml^−1^ *P. fluorescens* PpR24 in sterile diet with three replicates. Error bars represent standard error of the mean (*N* = 3).

Aphid mortality upon bacterial challenge may result from toxic shock produced by the bacteria or alternatively through profuse bacterial growth within the aphids. To test this, bacteria‐infected aphids were macerated at six time points and the resulting homogenate diluted and plated onto LB agar to enumerate bacteria. Trials were conducted with the most virulent bacterium from earlier trials, *P. fluorescens* PpR24 strain on *M*. *persicae* clone 4106A. Growth of PpR24 within 4106A was assessed through CFU enumeration every 12 h for three days following an initial inoculum load of 10^2^ CFU ml^−1^ in treated sachets. No PpR24 cells were recovered in the first 24 h (Fig. 3), while at 36 h the cell titre reached 2 × 10^4^ CFU aphid^−1^ increasing to 2 × 10^7^ CFU aphid^−1^ at 72 h. No bacteria were recovered from control aphids fed on non‐inoculated sachets.

**Fig. 4:**
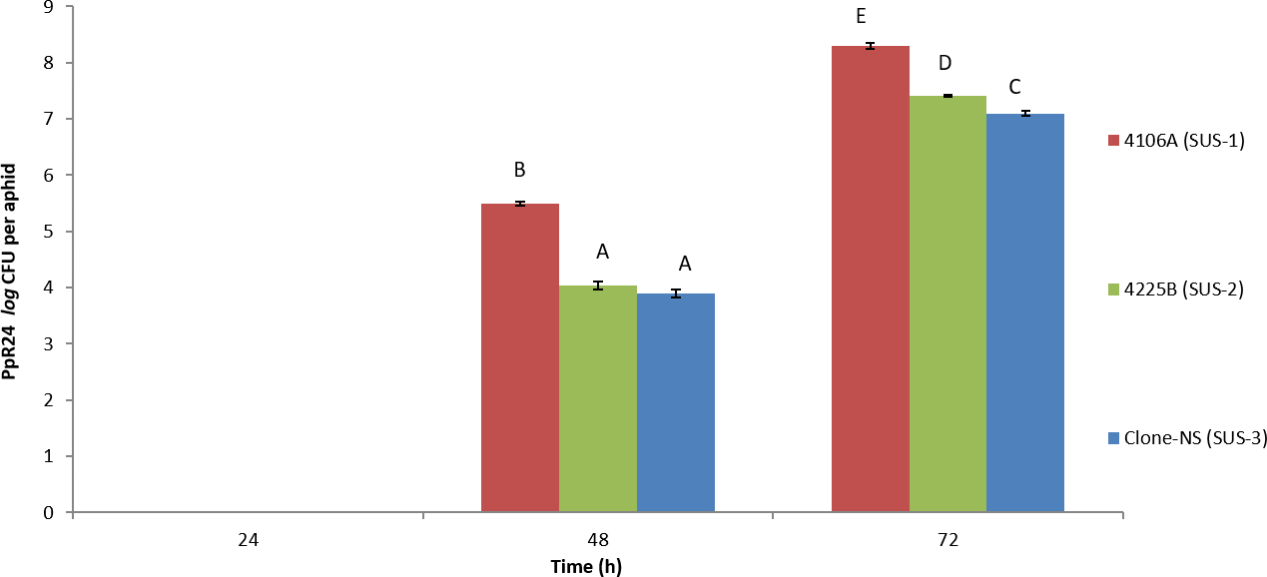
Assessment of *Pseudomonas fluorescens* PpR24 population in all infected insecticide‐susceptible aphid clones. Growth assay with inoculation dose of 10^2^ CFU ml^−1^ on all sensitive clones (*N* = 3) for three days. After 48 h*, P. fluorescens* PpR24 CFUs of each aphid clone were determined by enumeration on LB‐Nitrofurantoin plates. No colonies were recovered from control sachets. The data represent the mean and standard error of three biological replicates of *P. fluorescens* PpR24 treated sachets that contained ten aphids of each clone. The results show a statistically significant (different letter) decrease in CFUs of both 4225B and clone‐NS as compared to 4106A clone (*P* < 0.05).

Comparative studies, following similar methodologies, on IS clones 4225B and Clone‐NS revealed they were more susceptible than 4106A to PpR24 despite PpR24 being able to grow to a higher level in 4106A (Fig. 4; Fig. S5). PpR24 cells were only recovered from infected aphid clones after 24 h. At 48 and 72 h, a statistically lower (*P* < 0.05) titre of PpR24 was observed for both 4225B and Clone‐NS upon comparison to 4106A (Fig. 4). Furthermore, no aphid deaths were recorded in the initial 48h. At 72 h, 60% and 45% respective mortality rates were observed in Clone‐NS (Fig. S5G) and 4225B (Fig. S5F) with only 16% mortality reported in 4106A (Fig. S5A). Further confirmatory steps, at each time point, using PCR and specific primers (TcaAF1 and TcaAR1) to amplify the *tcaA* toxin gene of PpR24 were conducted to confirm re‐isolation of the inoculated strain.

These results indicate that, over an extended timeframe, consumption of low doses of bacterial cells may be sufficient to cause mortality to aphids.

### *Pseudomonas fluorescens* PpR24 survival *in planta*

**Fig. 5:**
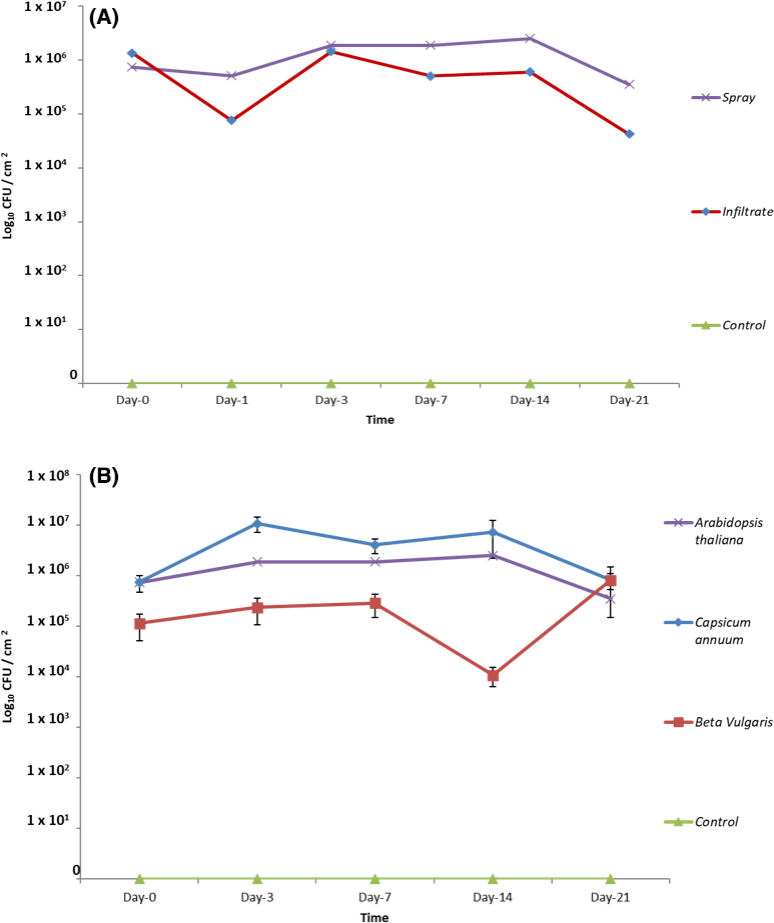
*Pseudomonas fluorescens* PpR24 colonization on *Arabidopsis thaliana*, *Capsicum annuum* and *Beta vulgaris*. (A) Bacterial populations recovered from *A. thaliana* leaves over a period of 21 days after spraying and infiltration with a cell suspension of 10^7^ CFU ml^−1^. For foliar spray, bacteria were suspended in sterile PBS solution and a leaf disc was collected at all time points. Each leaf disc (*N* = 6) was homogenized in PBS solution and serial dilutions were plated on LB with nitrofurantoin to count bacterial populations. The data presented are the mean and standard error of six biological replicates. (B) Bacterial populations were recovered from plant leaf surfaces over period of 21 days after spraying with cell suspension of 10^7^ CFUmL^‐1^. For the foliar spray, bacteria were suspended in sterile PBS solution and a leaf disc was collected at all time points. Each leaf disc (*N* = 6) was homogenized in PBS solution and serial dilutions were plated on LB with nitrofurantoin to count bacterial populations. The data presented are the mean and standard error of six biological replicates.

To examine the ability of PpR24 to survive on and inside plant leaves, bacterial colonization assays were conducted to examine survival rate of these bacteria on the surface of *Arabidopsis thaliana* (Col‐0 ecotype), *Beta vulgaris* and *Capsicum annuum* leaves. In a preliminary trial, we tested two methods of leaf inoculation (infiltration and spray). For both methodologies, we recovered a similar number of CFUs immediately following inoculation (time point 0). Following initial drops in counts in the first 24h for both methods, CFU counts were significantly higher in leaves which had been sprayed compared with those subjected to infiltration (Fig. 5A). Thus, foliar sprays were used in all subsequent assays. No bacteria were recovered from control plants in either method and for the duration of the experiment.

Following foliar spray inoculation of all three test plant species, bacterial survival was assessed at six time points: 0, 1, 3, 7, 14 and 21 days. Whole leaves were removed aseptically at each time point and processed to enumerate bacteria. PpR24 CFU counts on *C. annuum* reached a peak within the first 2–3 days and then slowly declined over the course of the 21‐day trials. With a sudden drop of PpR24 CFU counts at day 14, the overall bacterial populations remained relatively stable on *B. vulgaris* plants over the 21 days of trials (Fig. 5B) whereas CFU counts on *A. thaliana* declined. An analysis of the respective PpR24 CFU counts, tested by one‐way ANOVA, revealed no significant differences across all time points (ANOVA, *P* > 0.05) suggesting plant species did not adversely affect the ability of the bacterium to survive on the leaf surface. Additionally, no hypersensitive response was observed across the time period suggesting PpR24 is not pathogenic towards the test plant species, a prerequisite for the use of this species as a biocontrol. This was further confirmed in a hypersensitive reaction (HR) test on tobacco whereby high dose inoculation of PpR24 did not cause an HR whereas the control test using *P*. *syringae* pv. *tomato* did (Fig. S6).

### Effect of *P. fluorescens* PpR24 leaf spray inoculation on aphid (4106A) populations

**Fig. 6:**
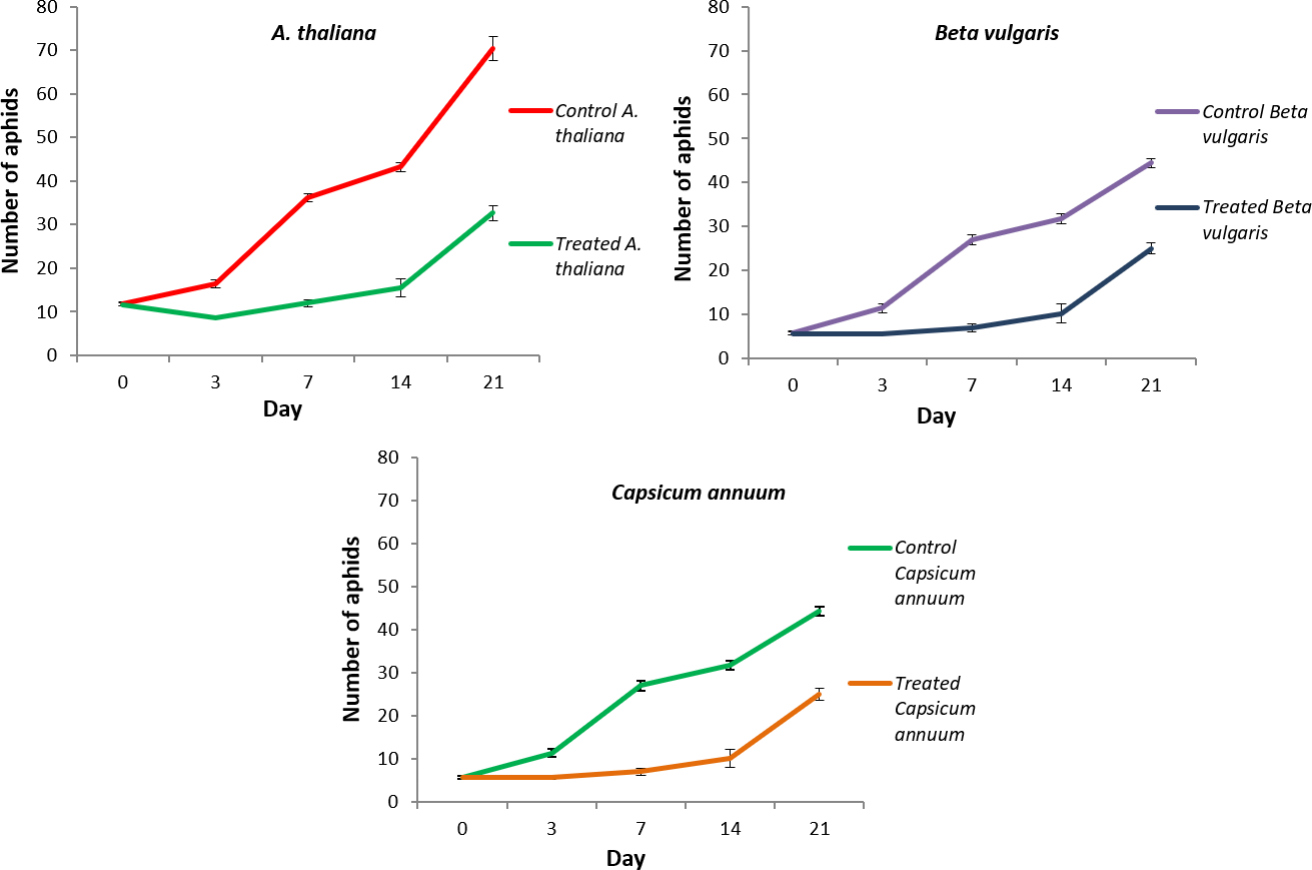
Effect on *M. persicae* (4106A) leaf populations after foliar application of *Pseudomonas fluorescens* PpR24 on different plants. Aphid populations (starting population of six aphids (*N* = 6) per plant at day‐0) were applied after bacterial spraying (when run‐off was achieved and the plants were allowed to dry for 4 h) and were recorded over a period of 21 days from non‐inoculated (control) and inoculated (treated) plants: *Arabidopsis thaliana*; *Beta vulgaris*; *Capsicum annuum*. The data presented are the mean and standard error of six biological replicates.

Biocontrol assays were conducted by transferring six adult aphids to previously inoculated (same day) PpR24 plant leaves and non‐inoculated controls. Aphid populations, consisting of both nymphs and adults, were enumerated over a 21‐day period. Aphid populations in all control plants grow exponentially. With significant differences in counts (ANOVA, *P* < 0.05) already detectable from day three, total aphid populations, after 21 days, were significantly lower in inoculated *A. thaliana*, *C. annuum* and *B. vulgaris* leaves, with respective final population counts being 57%, 68% and 69% smaller than control populations (Fig. 6).

To examine the period of protection provided by PpR24 following application, killing efficacy was assessed on *C. annuum* by introducing six aphids at 0, 3‐, 7‐, 14‐ and 21‐day post‐spraying. The aphid counts were recorded 28 days after the introduction of the aphids to the plant to examine the percentage control at different time intervals in relation to the aphid infestation level of control plants (Table 3). PpR24 provided excellent control of aphids with a 61‐88% efficacy rate after foliar application at all assessment intervals. The 88% efficacy control rate observed at 7 days after application was significantly higher than other time intervals (*P < 0.01).

**Table 3:** Summary of *Pseudomonas fluorescens* PpR24 efficacy trials to control *M. persicae* (4106A) aphid on *C*. *annuum* in response to different time intervals between PpR24 application and aphid infestation.

Aphid inoculation day	Aphid populations (Mean ± SE) on control plants after 28 days	Aphid populations (Mean ± SE) on Treated plants after 28 days	Aphid killing efficacy rate (%) at 28 days (Mean ± SE)
Day 0	648.8 ± 19.41	195.75 ± 7.05	69.80 ± 0.80
Day 3	473.25 ± 13.14	184 ± 2.04	61.04 ± 0.72
Day 7	749 ± 17.97	82.5 ± 8.19	88.86 ± 1.40
Day 14	521.75 ± 11.61	128 ± 15.05	75.63 ± 2.33
Day 21	852 ± 16.9	235 ± 8.22	72.39 ± 0.73

Aphid populations (starting population of six aphids (*N* = 6) per plant at day 0, 3, 7, 14 and 21) were recorded over a period of 28 days from non‐inoculated (control) and inoculated (treated) plants. The aphid‐killing efficacy rate was calculated (Abbott, 1925) after 28 days. The data presented are the mean and standard error of four biological replicates.

## Discussion

Insecticide resistance in aphids presents a major constraint on our ability to protect the yield and quality of several important crop plants. Because there are only limited numbers of insecticides with differing modes of action available, and as ongoing EU legislation is likely to place further limits on chemical insecticides, there is an urgent need to develop alternative control strategies. In this context, the interactions between insects and microorganisms could be of crucial importance as their study could lead to the discovery of novel biological molecules with the capacity to control pest species, as exemplified by the development of *B. thuringiensis* for insect control (Schnepf *et al*., 1998). There is evidence of phytopathogenic bacterial epiphytes including *Erwinia aphidicola* (Harada and Ishikawa, 1997), *P. syringae pv*. *syringae* (Stavrinides *et al*., 2010), *Pantoea stewartii* (Stavrinides *et al*., 2010) and *D. dadantii* (Grenier *et al*., 2006), being entomopathogenic, and particularly active against the pea aphid. Several phytopathogenic bacterial strains are thought to have initially exploited insects as vectors and over time evolved novel modes of interaction with insects, retaining an ability to colonize them and use them as secondary hosts (Nadarasah and Stavrinides, 2011).

In this study 140 bacterial strains were isolated from the phylloplane and rhizosphere of a range of plants. Nine of these exhibited promising yet variable degrees of aphicidal activity against *M. persicae*, and five other aphid species (Fig. S2). Other more established biopesticides are already known to infect a range of closely related species. For example, *B*. *thuringiensis* produces toxin proteins that are specific to, yet affect all, insect species within a specific clade or family (Höfte and Whiteley, 1989). Interestingly, our results revealed variable sensitivity between the trialled aphid species, with *B*. *brassicae* appearing to be particularly susceptible (Fig. S2). This suggests that some aphid species may be especially vulnerable to bacterial‐based biocontrol. Further testing of six of these bacterial isolates against several IR and IS clones of *M. persicae* revealed that *P. fluorescens* PpR24 had the greatest overall efficacy resulting in 90–100% mortality within 72 h at 10^7^ CFU ml^‐1^ (Fig. S5). While rapid cell concentration‐dependent decreases in toxicity were observed, it is notable that PpR24 was still 50% effective at 10^5^ CFU ml^−1^. The phylogenetic relationship of PpR24 with other pseudomonads indicated the closest fully sequenced relative is *P*. *fluorescens* SS101 (Fig. S1), which was also isolated from the rhizosphere of a crop plant (wheat).

The high efficacy of this plant‐derived *Pseudomonas* sp. against aphids is consistent with previous studies which found that an epiphytic strain of *P*. *syringae*, Cit7, can orally infect and kill both aphids and whiteflies within 72 h (Smee *et al*., 2017), and the bean pathogen *P. syringae* pv. *syringae* B728a can kill pea aphids in < 2 days (Stavrinides *et al*., 2009). Furthermore, *P. fluorescens* and *Pa*. *agglomerans* were previously identified as potential pathogens for *M. persicae* (Hashimoto, 2002). Only a few bacterial strains displayed a longer time to establish infection and caused 20–100% mortality in the various species within 72 h (Figs S2 and S3). These results are similar to the findings seen for *Dickeya dadantii* A428 strain and other enteric bacteria, which resulted in 50–100% aphid mortality after 4–5 days of ingestion of bacteria through the diet (Grenier *et al*., 2006).

It is often suggested that the mechanisms underpinning insecticide resistance in *M*. *persicae* can carry fitness costs in the absence of insecticides (Foster *et al*., 1997, 2000, 2003; ffrench‐Constant and Bass, 2017). Any such reduction in fitness might make insecticide‐resistant clones more susceptible to biocontrol agents. Alternatively, it is possible that mechanisms of resistance to insecticides could confer a degree of cross‐resistance to biocontrol agents with entomopathogens. For example, some resistance mechanisms have been shown to provide protection against oxidative stress, and this may provide broad protection to a range of xenobiotics (Vontas *et al*., 2001). Whilst significant variation was observed in the response of three IS and four IR clones to bacterial challenge no single bacterial strain was identified that was consistently more toxic to IR clones than IS clones. This suggests there is no fitness cost attributed to resistance (as a result of target‐site mutations or increased production of detoxifying enzymes) that makes such clones more susceptible to bacterial challenge. Further screening of a larger number of aphid genotypes carrying different resistance mechanisms should be conducted to verify that our findings were not influenced by the relatively small sample size employed in this study. Indeed, the two clones, 5444B and 5191A, exhibiting highest insecticide resistance, showed moderate levels of tolerance to *E. xiangfangensis* strain ER93, *Pa*. *agglomerans* PaR38 and *Enterobacter* CWR94, with up to 11‐fold differences in resistance compared with the IS clone 4106A (Table 2). Furthermore, 5444B also showed some resistance (fivefold) to both *Pseudomonas* strains. These findings are consistent with the hypothesis that enhanced production of detoxification enzymes in these aphid clones (or altered insecticide target sites) provides low‐level cross‐resistance to bacterial challenge. The aphid susceptible clone 4106A has been used as a standard control to measure baseline susceptibility or relative resistance of resistant clones for each bacterial challenge. However, due to differences in physical parameters such as water content, humidity and light source at the different laboratories (University of Reading and Rothamsted Research insectary), these variations may explain the differences in LC50 of 4106A clone being observed (Table 2). Additionally, another UK origin susceptible clone 4225B showed similar LC50 values as 4106A clone for all the different bacteria challenges apart from PpR24, which provides further evidence that there is no consistent correlation of insecticide resistance status and susceptibility to bacterial challenge. In contrast, an additional susceptible Clone‐NS showed large variation in LC50 values as compared with 4106A clone suggesting the genetic background is a more crucial factor in bacterial sensitivity than insecticide resistance status. Enumeration of PpR24 cells was done in three different infected susceptible aphid clones and observing this in relation to mortality rates showed an inverse correlation between 4106A versus 4225B and NS clones whereby the latter two clones could be killed at a higher rate with less bacterial cells. This suggests that there are distinct differences in the susceptibility of different aphid clones and are unlinked to differences in insecticide resistance. Indeed, the lack of a consistent trend in the response of IR and IS clones to bacterial challenge suggests this is a more likely explanation.

Regardless, the patterns and level of variation in IR and IS *M. persicae* clones observed are important for two reasons. First, the different susceptibility patterns of distinct genotypes of a single aphid species to bacterial challenge has implications for the application rates of any biological control based on the deployment of these bacteria or their toxin(s). Second, the differences in levels of tolerance (i.e. < 11‐fold between certain IR and IS clones) observed are relatively modest and application rates could be devised that would still ensure good efficacy against more tolerant aphid clones. This means that these biological control agents would be insecticide‐resistant ‘breaking’ (i.e. be able to still target insectide‐resistant clones where chemicals can not) and may provide a useful IPM tool and control option against populations of *M. persicae* that can no longer be controlled with conventional insecticides.

In defining appropriate application rates of bacterial biocontrol, it is useful to understand their capacity to replicate in the host. In this regard, we demonstrate the growth of PpR24 inside aphids following ingestion of cells suggesting it can successfully colonize and replicate within the aphid gut. These results are consistent with previous studies on two pathogenic *Pseudomonas* strains (*P. entomophila* L48 *and P. syringae* B728a), which were able to efficiently colonize and multiply inside the insect (lepidopteran) digestive tract ultimately killing the insect (Vodovar *et al*., 2005; Stavrinides *et al*., 2009). Importantly, PpR24 displayed potent insecticidal activity upon oral ingestion, when delivered in both artificial diet and via spray‐treated leaves. Indeed, foliar sprays of PpR24 successfully reduced aphid populations by an average rate of 55% on *A. thaliana*, *C. annuum* and *B. vulgaris* over a three‐week period (Fig. 6). Moreover, the average bacterial population of 2 × 10^7^ CFU cm^−2^ remained relatively stable over the 3 weeks without the induction of a plant hypersensitive response. This is important in indicating the bacterium does not appear to have the potential for causing plant disease. These results are consistent with earlier findings of root‐colonizing biocontrol strains, like *P. protegens* and *P. chlororaphis,* which were shown to display potent oral insecticidal activity and plant growth‐promoting traits (Ruffner *et al*., 2013; Flury *et al*., 2016). Our results are also supported by research which successfully demonstrated that a foliar spray of *P. fluorescens* at 1% controlled a cotton aphid (*Aphis gossypii)* infestation (Manjula *et al*., 2017). We showed that the highest rate of efficacy of control by PpR24 (86%) was achieved seven days after PpR24 application (Table 3). Nauen *et al*. (2015) recorded similar temporal findings in the control of aphids and whiteflies through use of a butenolide insecticide. The insecticide flupyradifurone showed excellent efficacy against various sucking pests, with different application methods and provided the highest level of control against lettuce aphids at 6–10 days after application, that is 96% efficacy. A particularly notable observation was the efficacy of the bacterium controlling the aphid population despite a potential lag phase from spraying the bacteria on plants versus the rapid growth of the aphid [at 21°C, *M*. *persicae* populations on sprouts can double in 3.1 days (van Emden, 1988)]. This may be attributed to effects on aphid fitness that have not yet been detected, thus requiring further examination. It will also be important to analyse whether the bacterium triggers any systemic resistance in the plant that may influence plant defence against the aphids.

In conclusion, the present work has identified a novel plant‐associated bacterium that may have applications as alternative means of aphid control in both agricultural and horticultural settings. During the formulation of bacterial‐based plant protecting products, insecticidal efficacy, bacterial longevity on plant surfaces, environmental safety and pest resistance to bacteria all need to be considered. We demonstrate that the efficacy of *P. fluorescens* PpR24 against the damaging aphid pest *M. persicae* is not compromised by pre‐existing resistance to chemical insecticides. Furthermore, we show that PpR24 survives on leaf surfaces for a period of at least three weeks whilst controlling aphid populations to an average of 55% on all tested plants. These data provide initial promise that *P. fluorescens* PpR24 may have utility in IPM strategies against *M. persicae*. Further work is now required to explore its effectiveness in a commercial setting, including: (i) *in planta* assays to establish minimum effective dosage rates which will reduce selection pressure and avoid resistance development (Hoy, 2008), (ii) testing the effect of polymeric additives, adjuvants, and surfactants on survival and stability, (iii) investigation of host specificity and impact on non‐target arthropods and beneficial insects such as ladybirds, (iv) investigate the variations of bacterial toxicity in the different aphid species and their implications on the survival and reproduction rate of aphids, and (v) investigation of the mechanism(s) of virulence. Past studies have implicated a Cry‐related toxin and bacterial aggregation in the gut, potentially causing occlusion, as potential mechanisms that cause aphid death, thus warranting further exploration (Stavrinides *et al*., 2010; Palma *et al*., 2014b).

## Experimental procedures

### Bacterial and aphid growth media and conditions

Bacteria were grown on one of three media at 27°C for 24 h (broth, with shaking) or 48 h on 1% (w/v) agar (Thermo Fisher Scientific, Scotland, UK) plates. Kings’ Medium B (KB, 1 l distilled H_2_O, Proteose peptone (Difco) 20 g, K_2_HPO_4_ 1.5 g, MgSO_4_.7H_2_O 1.5 g, glycerol 10 ml) (King *et al*., 1954), Lysogeny Broth (LB, 1 l distilled H_2_O, Bacto‐Tryptone (Oxoid) 10 g, Bacto‐yeast extract (Oxoid) (Oxoid Limited, Hampshire, UK) 5 g, NaCl (BDH) (BDH laboratory supplies, Dorset, UK) 10 g, Glucose (BDH) 1 g) (Miller, 1972) and M9 minimal medium (M9, Na_2_HPO_4_ 33.91 g; KH_2_PO_4_ 15 g; NaCl 2.5 g; 2 ml 1 M MgSO_4_·7H_2_O; 100 µl 1 M CaCl_2_·6H_2_O; 20 ml 20% Glucose; 10 ml 100 mg ml^−1^ NH_4_Cl) were used for culturing the bacterial strains. Stock solutions of Nitrofurantoin 100 µg ml^−1^ were prepared in dimethyl sulfoxide solvent and used as a selective agent for pseudomonads. Different aphid species were reared on various host plants as detailed in Table S4. The *M. persicae* clones used in this study, carrying different combinations of insecticide resistance mechanisms, are detailed in Table S5. Clones were originally established from individual ancestral females, collected at different times from widely dispersed populations located in the United Kingdom and mainland Europe.

Aphids were reared in two different ways in this study.

#### Leaf box rearing

Asexual forms of each *M. persicae* clones were maintained in the laboratory on excised leaves in small plastic box‐cages (Blackman, 1971), at 21°C, under a long day (16‐h light/8‐h dark) regime. To set up new generations of each clone, six apterous young adults were moved to each box (using a wetted fine paintbrush, size‐3) and left them to generate approximately 15 nymphs over the course of 2–3 days. Parents were then removed leaving age‐synchronized aphid cohorts that could be utilized in bioassays once they reached adulthood.

#### Cage rearing

Cage rearing was used to generate large aphid populations. Each clone was reared parthenogenetically in an insect cage on 4‐week‐old Chinese cabbage plants under a 21°C, long day (16‐h light/8‐h dark) regime. New generations of each clone were set up by inoculating plants with aphid populations established for 2 weeks in leaf boxes and leaving them to produce adults for up to 4 weeks. Similarly, other aphid species were reared on their appropriate plant host species listed in Table S4.

All UK‐native insecticide‐resistant (IR) and insecticide‐susceptible (IS) aphid bioassays were performed in insect containment rearing rooms at the University of Reading. Bioassays conducted on non‐UK originating aphids were carried out at the specialist containment insectary of Rothamsted Research (Harpenden, UK). All physical and environmental parameters for aphid bioassays, including aphid rearing, growth conditions and inoculation protocols were replicated at both sites to minimize variation.

### Isolation of bacteria

A list of plant species with either no known aphid pests or having the ability to actively deter aphids was obtained from the late Dr V.F. Eastop (Natural History Museum, London) and used as the basis for sampling. Three sets of samples of leaf, root and soil were taken from ten different individual plants (Table S1) per species found at seven different locations on the University of Reading Whiteknights campus, Reading, UK (51.4412, −0.9414), its commercial glasshouses and private gardens (Table S1). Samples were collected aseptically using sterilized metal forceps, scissors and trowels and placed into sterile 50 ml polypropylene falcon tubes, returned to the laboratory and placed in a fridge at 4°C before further processing. For each leaf, root and soil sample, 1 g was weighed and macerated in 500 μl of PBS (8 g NaCl, 0.2 g KH_2_PO_4_, 2.9 g Na_2_HPO_4_.12H_2_O, 0.2 g KCl l^−1^ H_2_O; pH 7.4). The samples were serially diluted in PBS and dilutions were spread plated onto various solid media (LB, KB and M9) to maximize the recovery of bacterial strains with varying nutritional requirements. Plates were incubated overnight at 27°C in the dark. Water samples from Whiteknights Lake were diluted to 10^−6^ CFU ml^−1^. Insect specimens were homogenized in 500 μl of sterile PBS using a sterile plastic mortar and pestle and further serially diluted to 10^−6^ CFU ml^−1^. For each dilution 10^−3^ to 10^−6^, 100 μl of suspension was spread in triplicate on to LB, KB and M9 agar plates. Individual colonies of distinct morphotypes were selected and re‐streaked onto new agar plates and incubated overnight at 27°C to obtain pure cultures. Cultures derived from single colonies were inoculated into 3 ml sterile LB broth, grown in a shaking incubator (200 rpm) for 12 h at 27°C and preserved in 20% (*v*/*v*) glycerol at −80°C. For subsequent laboratory work, all purified strains were routinely maintained on LB agar medium.

### Aphid toxicity assay

Purified environmental isolates were initially screened through 10 adult *M. persicae* (standard UK origin *M. persicae* 4106A clone) and the best aphid‐killing isolates re‐screened through 30 adult *M. persicae* to test for pathogenicity. To maintain sterility and avoid contamination, all work was conducted in a laminar flow hood. The aphid mortality assay was composed of the preparation of a specialist aphid feeding diet and inoculation of bacteria into the diet.

#### Preparation of aphid feeding sachets

Sachets of diet sandwiched between two sterile surfaces of Parafilm^®^ (Bemis, USA) on Perspex^®^ cylinders (25 mm depth, 25 mm internal diameter) were prepared following the procedures of van Emden and Wild (2020) and 10–15 aphids were transferred from maintenance plants into each test cylinder using a fine paintbrush. The bottom end of the cylinder was covered with the final square of Parafilm. The diet recipe is given in Table S6 (van Emden and Wild, 2020).

#### Inoculating the diet with bacterial strains

The bacterial strains were recovered from −80°C and single colonies grown in LB at 27°C for 12–15 h. The microbial cell density was determined using a spectrophotometer and then normalized to an OD_600_ of 1. This corresponds to a concentration of approximately 10^9^ colony‐forming units (CFU) ml^−1^. Cells were washed three times and re‐suspended in 10 mM MgCl_2_ and mixed with the Mittler diet after it had been passed through a disposable bacterial filter during sachet preparation, at a final microbial concentration of 10^7^ CFU ml^−1^. Control sachets containing sterile diet amended with 10 mM MgCl_2_ alone were prepared alongside.

For the preliminary screening of aphid‐killing bacteria, a single dose of 10^7^ CFU ml^−1^ was used in the aphid toxicity assay. Three replicates of 600 μl of Mittler diet containing bacteria were introduced in the standard parafilm sachets. 10–15 adult aphids were placed on each sachet and aphid mortality readings were recorded at 24, 48 and 72 h. All aphid sachets were maintained under the same environmental conditions described for aphid colonies. Even in *in vitro* conditions, few nymphs were produced over the period of observation, but final aphid death counts represented only adult deaths. An aphid was counted as dead if turned brown and/or was observed at the bottom of the cylinder in a non‐moving state. Live aphids were most generally observed feeding at the underside of the parafilm abutting the diet. Bacterial strains were classed as pathogenic to the aphids if aphid death was triggered during the first 48h of observation. No death was observed in the control sachets.

Further detailed assessment of aphid mortality on different aphid clones {insecticide‐resistant (IR) and insecticide‐susceptible (IS) listed in Table S5} with the six best aphid‐killing bacteria was performed. We carried out the previously described aphid toxicity assay with infecting doses ranging from 10^7^ to 10^2^ CFU ml^−1^ for three days. Each bacterial treatment with different doses was replicated three times with 10–15 adult aphids per replicate over course of all experiments. Data obtained from these bioassays were used for determination of the LC_50_ value of all aphid clones for their individual bacterial treatment. Standarization of the bioassay was performed on the standard UK origin susceptible clone 4106A; therefore, this clone is considered as the reference aphid clone for calculating the resistance ratio in different physical laboratory conditions.

### 16S rRNA gene sequencing for bacterial identification

The bacterial strains that were shown to have a pathogenic effect on *M. persicae* were identified by sequence analysis of the 16S rRNA gene. Colony PCR was used to amplify this gene using a Techne Thermal Cycler and the universal 16S rRNA primers 8F (5′‐AGAGTTTGATCCTGGCTCAG‐3′) and 1492R (5′‐GGTTACCTTGTTACGACTT‐3′) as described by Singh *et al*. (2013). Each PCR reaction mixture was prepared as follows: 10 μl 5× Phusion HF buffer; 1 μl 10 mM dNTPS; 1 μl of each 10 μM forward and reverse primer; 0.5–1 μl template; 0.5 μl Phusion polymerase (1 unit/50 μl); molecular biology grade water to 50 μl. PCR cycling conditions were 95°C for 5 min, 30 cycles of 95°C for 30 s, 58°C for 30 s, 72°C for 1 min and a final extension at 72°C for 5min. PCR products were purified using the Genomic DNA Clean and Concentrator™‐25 kit (Zymo Research, Irvine, USA) according to manufacturer’s instructions, and forward and reverse strands sequenced by Source BioScience UK Limited, Oxford. Sequences were aligned and the resulting consensus read compared with the 16S rDNA sequences in the National Center for Biotechnology Information (NCBI) database (http://www.ncbi.nlm.nih.gov/BLAST/) using Basic Local Alignment Search Tool (BLAST).

Evolutionary relationships between *Pseudomonas* strain PpR24 and their closest genetically related species were investigated using a Multilocus sequence typing (MLST) approach developed by Andreani *et al*. (2014) to characterize the *P. fluorescens* group. The seven MLST loci sequences *glnS, gyrB, ileS, nuoD, recA, rpoB and rpoD* from 97 strains (Andreani *et al*., 2014) were downloaded from NCBI (January 2017), while those from genomes sequenced in this study were extracted by blasting the MLST sequences of the reference genome *P. fluorescens* A506 against the genomes. This dataset was enriched with the MLST sequences extracted from the 79 genomes of the *P. fluorescens* species and most related species gathered in the genetic cluster 2 (Monteil *et al*., 2016) in which all loci were detected (using a BLAST word size of 11 pb, a minimum sequence identity of 70% and alignment length of 50%). Gene sequences were aligned independently using MUSCLE and then concatenated into a single alignment of 3541 bp among which 1428 sites were polymorphic. A maximum likelihood (ML) tree was built with RAxML 8.2.6 (Stamatakis, 2014) under the GAMMA model of rate heterogeneity using empirical nucleotide frequencies and the GTR nucleotide substitution model. A total of 249 bootstrap replicates automatically determined by the MRE‐based bootstrapping criterion were conducted under the rapid bootstrapping algorithm, among which 100 were sampled to generate proportional support values.

### Bacterial plant colonization assay

For plant bioassays Chinese cabbage (*Brassica napus* L. var *chinensis* cv. Wong Bok) (Simply Seed, Nottingham, UK), organic red sweet pepper Sapporo (RZ) (*Capsicum annuum* L.) (Rijk Zwaan UK Ltd, York, UK), sugar beet (*Beta vulgaris*) and *Arabidopsis thaliana* (Col‐O ecotype) were used. Plant seeds were grown in Clover seed modular compost (Clover quality peat product, County Tyrone, North Ireland) containing peat, sand and wetting agents at 75% humidity, light intensity of 150 μmol m^2^ s^−1^ (16 h photoperiod: day temperature of 22°C, night temperature of 20°C).

#### Foliar spray method

To acclimatize plants to the physical parameters of the growth chambers (22°C, 75% Rh, 16/8‐h light/dark cycle), plants were moved three days prior to bacterial inoculation bioassays. *P. fluorescens* PpR24 was grown as described above and cultures were washed twice with sterile PBS and re‐suspended in fresh PBS to an OD_600_ of 1.5 ml. Bacterial suspension in the PBS was applied as foliar sprays to ‘run‐off’ on both the adaxial and abaxial sides of leaves of 3‐week‐old plants using a hand atomizer (Buerkle™, Fisher Scientific, England, UK). The same volume of sterile PBS was sprayed onto un‐inoculated control plants. After spraying, plants were allowed to dry in a sterile flow cabinet. On days 0, 1, 3, 7, 14, 21 and 28, 0.28 cm^2^ sections of infected and control leaves were aseptically removed using a sterile steel core borer and transferred to sterile microcentrifuge tubes containing 200 μl PBS. Leaf samples were thoroughly homogenized using sterile plastic pestles. A dilution series (10^0^–10^‐3^) was prepared per sample and aliquots plated onto LB agar with Nitrofurantoin (100 µg ml^‐1^) in triplicate. Plates were incubated O/N at 27°C and colonies were counted for each sample to calculate CFUs per leaf area.

#### Leaf infiltration method

As in foliar spray trials, three‐week‐old plants were moved to growth chambers (set at 22°C, 75% Rh) to acclimatize for 3 days prior to infiltration. For each treatment, bacterial suspensions were prepared as described above. A sterile 200 μl yellow pipette tip was used to puncture a small hole in the abaxial side of the leaf. A 1 ml sterile plastic syringe (Terumo, Belgium) containing the bacterial suspension in PBS was pressed against the hole and a small amount of suspension infiltrated into the plant leaf. This procedure was repeated on other punctured areas of the leaf tissue until 1 ml of total bacteria culture was infiltrated into the leaf. Control leaves and plants received 1 ml of sterile PBS. Plants were dried in a sterile flow cabinet. At each time point, plants were removed from the pots, inoculated leaves excised and placed into sterile microcentrifuge tubes and processed as previously. Bacterial enumeration at all time points represents total counts, that is both for external surface and internal bacteria populations.

### *In* *planta* bacterial biocontrol of aphids

For *P. fluorescens* PpR24 *in planta* trials, apterous young adult *M. persicae* IS clone 4106A was used to evaluate biocontrol efficacy. Three‐week‐old *A. thaliana* Col‐0, *B. vulgaris* and *C. annuum* plants were spray inoculated with 10^7^ CFU ml^‐1^ PpR24, or water control, until run‐off was achieved and the plants were allowed to dry for 4 h. Six adult aphids were introduced on the bacteria‐inoculated and non‐inoculated plant species on the same day of bacterial inoculation (Day 0). The aphid counts, which represented both nymphs and adults, were recorded twice weekly as accumulated counts on control and treated plants for 3 weeks.

### Statistical analysis

All statistical analysis was conducted in GenStat version 16.0 for Windows (VSN International Ltd, Hemel Hempstead, UK). Data sets of IR *M. persicae* clones mortality at 48 and 72 h time points were analysed by two‐way ANOVA, with Tukey–Kramer HSD test to determine significant difference between treatment groups. The mean values that were significantly different (*P* > 0.05) by this test are indicated by the different letters in figures. General analysis of variance was also applied to the data from the bioassay of different IR *M. persicae* clones at 72 h to study main effects and interactions of the various parameters **(**bacterial strain, dose and aphid clone**)** on the mortality. In this analysis, the 72 h aphid mortality of 4106A aphid clone (University of Reading laboratory) compared with the other four IR and two IS aphid clones were considered.

To calculate bacterial LC_50_ values of each aphid clone, 72 h aphid mortality readings at six bacterial concentrations ranging from 10^7^ to 10^2^ CFU ml^−1^ were transformed to mortality probits, which produced a line of regression. This linear relationship was imported into GenStat and through use of ‘Probit analysis tool’, logs of explanatory variables (log concentration of bacteria) and number of responding (mortality probits) relationships were analysed. The 95% confidence limits were used to compare the LC_50_ between the bacterial treatment groups. Differences were considered non‐significant if their 95% confidence values overlapped (Forrester *et al*., 1993).

For CFU calculations, data were transformed to log10 for statistical analysis and graphical presentation, and analysed by ANOVA with the Tukey MCT in GenStat version 16.0 for Windows (VSN International Ltd, Hemel Hempstead, UK).

For biocontrol assays, aphid‐killing efficacy rate was calculated by Abbott (1925) formula = (Aphid population on control plants – Aphid population on treated plants) / Aphid population on control plants * 100.

## Conflict of interest

All authors declare there is no conflict of interest.

## Supporting information


**Table S1**. Sample origins and their locations used for microbial isolation.
**Table S2**. Statistical similarities and differences between 72 h aphid mortality caused by various bacterial strains when ingested by different aphid species.
**Table S3**. Summary of General Analysis of variance for aphid mortality at 72 hours in relation to bacterial strains, aphid clones and infection doses and their interaction between all test parameters.
**Table S4**. Aphid species and their host plants used in this study.
**Table S5**. *Myzus persicae* clones included in the study and their insecticide resistance mechanisms.
**Table S6**. Composition of the Mittler aphid artificial diet.
**Fig. S1**. Maximum Likelihood (ML) tree of 177 *Pseudomonas fluorescens* related strains based on the MLST scheme of Andreani et al. (2014) rooted with *Pseudomonas aeruginosa* strain PAO1. Trees were drawn to scale and branch length represents the number of base substitutions per site. Nodes annotated with a circle are supported by bootstraps values superior to 70%. The scale bar represents the number of substitutions per site. *P. fluorescens* PpR24 and *P. fluorescens* PfR37 are shown lower left in bold.
**Fig. S2**. Differential killing effects of plant‐associated bacteria on different aphid species. Mortality assay showing the percentage of dead aphids (*N* = 10) (A) *Aphis fabae*, (B) *Brevicoryne brassicae*, (C) *Macrosiphum albifrons* (D) *Nasonovia ribsnigri*, (E) *Aulacorthum solani* at 72 hours after ingestion of artificial diet inoculated with various bacterial cells (10^7^ CFU ml^−1^). Error bars represent standard error of the mean of three biological replicates. Bacterial strains tested ‐ *Acinetobacter* sp. AjR35, *Enterobacte*r sp. CwR94, *Enterobacter* sp. ER93, *Pantoea* sp. PaR8, *Pantoea agglomerans* PaR38, *Pseudomonas fluorescens* PfR37, *Pseudomonas fluorescens* PpR24, *Pseudomonas* sp. PR10 & *Pseudomonas rhizosphaerae* PrR91. ANOVA detected statistically significant differences (*P* < 0.05) at 72 hours and comparison of means by Duncan's multiple comparisons to the control were shown as letters (where different letters on the graphs indicate statistically significant differences) shown in table S2.
**Fig. S3**. Assessment of aphid (*Myzus persicae*) mortality by various bacterial species. Mortality assay showing the percentage of dead aphids (*N* = 10) at 72 h after ingestion of artificial diet inoculated with various bacterial cells (10^7^ CFU ml^−1^). Control: Ten aphids were fed in sterile diet with three replicates. Error bars represent standard error of the mean of three biological replicates. ANOVA detected statistically significant differences (*P* < 0.05) and comparison of means by Tukey‐Kramer HSD were shown as letters (where different letters on the graphs indicate statistically significant differences). Aphid clones:Three susceptible clones “4106A‐SUS 1”, “4225B‐SUS 2” & “Clone‐NS SUS‐3” and four resistant clones “New green – RES 1”, “794J2 – RES 2”, ”5191A – RES 3” and “5444B – RES 4”. *Note‐ Reference clone 4106A 72‐hour mortality readings from Figure 1. were used for comparison. Bacterial strains tested:*Pseudomonas fluorescens* PpR24, *Pantoea agglomerans* PaR38, *Enterobacter* sp. CwR94, *Pantoea* sp. PaR8, *Pseudomonas fluorescens* PfR37, *Enterobacter* sp. ER93, *Pseudomonas rhizosphaerae* PrR91, *Pseudomonas* sp. PR10 & *Acinetobacter* sp. AjR35.
**Fig. S4**. Effect of bacterial concentration on aphid mortality for various aphid clones after 48 h. Three different experiments were carried out based on the availability of growth rooms, with clone 4106A used as a common comparator: Set I Aphid rearing room (University of Reading), Set II Specialist containment Insectary, (Rothamsted Research) and Set III Controlled growth cabinet (University of Reading). Aphid mortality assay showing the percentage (*N* = 10) of dead aphids{(A) 4106A (SUS‐1), (B) New green (RES‐1), (C) 794J2 (RES ‐2), (D) 5191A (RES ‐3), (E) 5444B (RES‐4), (F) Clone 4225B (SUS‐2), (G) Clone NS (SUS‐3)} after ingestion of artificial diet inoculated with various bacterial species cells at 1 x 10^5^ CFU ml^−1^ (green bars), or 1 x 10^6^ CFU ml^−1^ (red bars), or 1 x 10^7^ CFU ml^−1^ (blue bars), for 48 h. No death was reported in control and lower concentration treated sachets. The data presented are the mean and standard error of three biological replicates. ANOVA detected statistically significant differences (*P* < 0.05) and comparison of means by Tukey‐Kramer HSD are shown as letters (different letters on the graphs indicate statistically significant differences). Bacterial strains tested ‐ *Pseudomonas* *fluorescens* PpR24, *Pseudomonas fluorescens* PfR37, *Pantoea* sp. PaR8, *Pantoea agglomerans* PaR38, *Enterobacte*r sp. CwR94 and *Enterobacter* sp. ER93.
**Fig. S5**. Effect of bacterial concentration on aphid mortality for various aphid clones after 72 h. Three different experiments were carried out based on the availability of growth rooms, with clone 4106A used as a common comparator: Set I Aphid rearing room (University of Reading), Set II Specialist containment Insectary, (Rothamsted Research) and Set III Controlled growth cabinet (University of Reading). Aphid mortality assay showing the percentage (*N* = 10) of dead aphids {(A) 4106A (SUS‐1), (B) New green (RES‐1), (C) 794J2 (RES ‐2), (D) 5191A (RES ‐3), (E) 5444B (RES‐4), (F) Clone 4225B (SUS‐2), (G) Clone NS (SUS‐3)} after ingestion of artificial diet inoculated with various bacterial species cells at 1 x 10^2^ CFU ml^−1^ (orange bars), 1 x 10^3^ CFU ml^−1^ (light blue bars), 1 x 10^4^ CFU ml^−1^ (purple bars), 1 x 10^5^ CFU ml^−1^ ^−1^ (green bars), or 1 x 10^6^ CFU ml^−1^ (red bars), or 1 x 10^7^ CFU ml^−1^ dark blue bars), for 72 h. No death was observed in control and lower concentration treated sachets. The data presented are the mean and standard error of three biological replicates. ANOVA detected statistically significant differences (*P* < 0.05) and comparison of means by Tukey‐Kramer HSD are shown as letters (different letters on the graphs) indicate statistically significant differences. Bacterial strains tested ‐ *Pseudomonas* *fluorescens* PpR24, *Pseudomonas fluorescens* PfR37, *Pantoea* sp. PaR8, *Pantoea agglomerans* PaR38, *Enterobacte*r sp. CwR94 and *Enterobacter* sp. ER93.
**Fig. S6**. Assessment of Hypersensitive response (HR) in peppers after foliar spray of different bacteria at 3 day post inoculation (dpi). Different bacterial suspensions in water at a concentration of 10^7^ CFU ml^−1^ were sprayed on pepper (*Capsicum annuum* cv. Sapporo (RZ)) plants: A. *P. syringae* pv. *tomato* DC3000‐ Positive HR response; B. *P. fluorescens* PpR24*‐* No HR; C. Control (water) No HR. At day 3, the yellow arrow indicates leaf showing HR. The numbers of individual symptomatic plants of the four plants per treatment are indicated.
