# Supplementary material for: Identification of novel aphid‐killing bacteria to protect plants
Source: Microb Biotechnol. 2021 Aug 1;15(4):1203–20. doi: 10.1111/1751-7915.13902 (PMC8966022; doi:10.1111/1751-7915.13902)
Supplement: Supplementary file 1 — Table S1. Sample origins and their locations used for microbial isolation. Table S2. Statistical similarities and differences between 72 h aphid mortality caused by various bacterial strains when ingested by different aphid species. Table S3. Summary of General Analysis of variance for aphid mortality at 72 hours in relation to bacterial strains, aphid clones and infection doses and their interaction between all test parameters. Table S4. Aphid species and their host plants used in this study. Table S5. Myzus persicae clones included in the study and their insecticide resistance mechanisms. Table S6. Composition of the Mittler aphid artificial diet. Fig. S1. Maximum Likelihood (ML) tree of 177 Pseudomonas fluorescens related strains based on the MLST scheme of Andreani et al. (2014) rooted with Pseudomonas aeruginosa strain PAO1. Trees were drawn to scale and branch length represents the number of base substitutions per site. Nodes annotated with a circle are supported by bootstraps values superior to 70%. The scale bar represents the number of substitutions per site. P. fluorescens PpR24 and P. fluorescens PfR37 are shown lower left in bold. Fig. S2. Differential killing effects of plant‐associated bacteria on different aphid species. Mortality assay showing the percentage of dead aphids (N = 10) (A) Aphis fabae, (B) Brevicoryne brassicae, (C) Macrosiphum albifrons (D) Nasonovia ribsnigri, (E) Aulacorthum solani at 72 hours after ingestion of artificial diet inoculated with various bacterial cells (107 CFU ml−1). Error bars represent standard error of the mean of three biological replicates. Bacterial strains tested ‐ Acinetobacter sp. AjR35, Enterobacter sp. CwR94, Enterobacter sp. ER93, Pantoea sp. PaR8, Pantoea agglomerans PaR38, Pseudomonas fluorescens PfR37, Pseudomonas fluorescens PpR24, Pseudomonas sp. PR10 & Pseudomonas rhizosphaerae PrR91. ANOVA detected statistically significant differences (P < 0.05) at 72 hours and comparison of means by Duncan's multip [file MBT2-15-1203-s001.docx]

**Supplementary information**

Tables

**Table S1 Sample origins and their locations used for microbial isolation.**

| **Sample origin** | **Common name** | **Latin name** | **Location** | **Sample taken** |
| --- | --- | --- | --- | --- |
| Plant | Maize | *Zea mays* | Experimental gardens | Leaf, root, soil. |
| Plant | Cabbage | *Brassica oleracea* | Experimental gardens | Leaf, root soil |
| Plant | Chilli pepper | *Capsicum annuum* | Experimental gardens | Leaf, root soil |
| Plant | Pepper | *Capsicum annuum* | Private garden | Leaf, root, soil |
| Plant | Tomato | *Solanum lycopersicum* | Private garden | Leaf, root, soil |
| Plant | Hammalias | *Hamamelidae, Urticales* | Harris gardens | Leaf, soil. |
| Plant | Magnolia | *Magnolia virginiana* | Harris gardens | Leaf, root, soil |
| Plant | Viola | *Viola arvensis* | Private garden | Leaf, root, soil |
| Invertebrate | Carabid beetle | *Broscus cephalotes* | Whiteknights lake | Whole organism |
| Plant | Strawberry | *Fragaria ananassa* | Experimental glass | Leaf, root, soil |
| Plant | Pepper (Cantelo) | *Capsicum annuum* | Cantelo nursery | Leaf, root, soil |
| Lake water | N/A | N/A | Whiteknights lake | Vial of water from edge of lake |

**Table S2** **Statistical similarities and differences between 72 h aphid mortality caused by various bacterial strains when ingested by different aphid species.**

| **Aphid Species /bacterial Strain** | ***Myzus persicae*** | | ***A.fabae*** | | ***B. brassicae*** | | ***M. albifrons*** | | ***N. ribsnigri*** | | ***A.solani*** | |
| --- | --- | --- | --- | --- | --- | --- | --- | --- | --- | --- | --- | --- |
|  | Aphid mortality % (72 h) | Significant letter | Aphid mortality % (72 h) | Significant letter | Aphid mortality % (72 h) | Significant letter | Aphid mortality % (72 h) | Significant letter | Aphid mortality % (72 h) | Significant letter | Aphid mortality % (72 h) | Significant letter |
| **Control** | 0 | A | 0 | A | 0 | A | 0 | A | 0 | A | 0 | A |
| **AjR35** | 20 | B | 26.67 | B | 70 | B,C | 13.33 | A | 70 | B,C,D | 90 | B,C |
| **ER93** | 63.33 | D | 26.67 | B | 53.33 | B | 20 | A | 40 | B | 66.67 | B |
| **PrR91** | 20 | B | 43.33 | C | 80 | C,D,E | 100 | B | 83.33 | C,D | 76.67 | B |
| **CwR94** | 46.67 | C | 46.67 | C,D | 96.67 | D,E | 13.33 | A | 56.67 | B,C | 86.67 | B,C |
| **PaR38** | 100 | F | 53.33 | C,D,E | 76.67 | B,C,D,E | 86.67 | B | 100 | D | 100 | C |
| **PR10** | 0 | A | 60 | D,E | 73.33 | B,C,D | 73.33 | B | 86.67 | C,D | 76.67 | B |
| **PaR8** | 90 | E | 63.33 | E | 93.33 | C,D,E | 100 | B | 70 | B,C,D | 70 | B |
| **PfR 37** | 100 | F | 100 | F | 90 | C,D,E | 93.33 | B | 56.67 | B,C | 73.33 | B |
| **PpR24** | 100 | F | 100 | F | 100 | E | 83.33 | B | 100 | D | 100 | C |
| Duncan's multiple comparisons compared means of aphid mortality between different tested bacterial species & control within each aphid species. Differences are shown as letters where different letters indicate statistically significant differences at the 72 hour time point. | | | | | | | | | | | | |

**Table S3 Summary of General Analysis of variance for aphid mortality at 72 hours in relation to bacterial strains, aphid clones and infection doses and their interaction between all test parameters.**

| **A. Set I Aphid rearing room (University of Reading)**  **Aphid clone tested - 4106A (SUS-1), New green (RES-1) & 794J2 (RES -2).** | | | | | |
| --- | --- | --- | --- | --- | --- |
| **Source** | **Degrees of Freedom (DF)** | **Sum of Squares (SS)** | **Means of square (MS)** | **Variance ratio** | **F-probability** |
| **Aphid clone** | 2 | 2415.43 | 1207.72 | 25.25 | <.001 |
| **Bacteria** | 5 | 180954.3 | 36190.86 | 756.51 | <.001 |
| **Dose** | 5 | 254724.7 | 50944.94 | 1064.91 | <.001 |
| **Aphid clone*Bacteria** | 10 | 4921.6 | 492.16 | 10.29 | <.001 |
| **Aphid clone*Dose** | 10 | 1106.79 | 110.68 | 2.31 | 0.013 |
| **Bacteria*Dose** | 25 | 84990.12 | 3399.6 | 71.06 | <.001 |
| **Aphid clone*Bacteria*Dose** | 50 | 16000.62 | 320.01 | 6.69 | <.001 |
| **Residual** | 216 | 10333.33 | 47.84 |  |  |
| **Total** | 323 | 555446.9 |  |  |  |
| **B. Set II**  **Specialist containment Insectary (Rothamsted research)**  **Aphid clone tested - 4106A (SUS-1), 5191A (RES 3) & 544B (RES -4).** | | | | | |
| **Source** | **Degrees of Freedom (DF)** | **Sum of Squares (SS)** | **Means of square (MS)** | **Variance ratio** | **F-probability** |
| **Aphid clone** | 2 | 2010.34 | 1005.17 | 32.49 | <.001 |
| **Bacteria** | 5 | 105326.3 | 21065.26 | 680.81 | <.001 |
| **Dose** | 5 | 202880 | 40576 | 1311.38 | <.001 |
| **Aphid clone*Bacteria** | 10 | 6285.03 | 628.5 | 20.31 | <.001 |
| **Aphid clone*Dose** | 10 | 3747.99 | 374.8 | 12.11 | <.001 |
| **Bacteria*Dose** | 25 | 60288.97 | 2411.56 | 77.94 | <.001 |
| **Aphid clone*Bacteria*Dose** | 50 | 7973.3 | 159.47 | 5.15 | <.001 |
| **Residual** | 216 | 6683.33 | 30.94 |  |  |
| **Total** | 323 | 395195.3 |  |  |  |
| **C. Set III Controlled growth cabinet (University of Reading)**  **Aphid clone tested - 4106A (SUS-1), 4225B (SUS-2) & Clone NS (SUS-3).** | | | | | |
| **Source** | **Degrees of Freedom (DF)** | **Sum of Squares (SS)** | **Means of square (MS)** | **Variance ratio** | **F-probability** |
| **Aphid clone** | 2 | 6304.32 | 3152.16 | 50.94 | <.001 |
| **Bacteria** | 5 | 205143.2 | 41028.64 | 663.01 | <.001 |
| **Dose** | 5 | 226580.3 | 45316.05 | 732.29 | <.001 |
| **Aphid clone*Bacteria** | 10 | 7684.57 | 768.46 | 12.42 | <.001 |
| **Aphid clone*Dose** | 10 | 2114.2 | 211.42 | 3.42 | <.001 |
| **Bacteria*Dose** | 25 | 59189.2 | 2367.57 | 38.26 | <.001 |
| **Aphid clone*Bacteria*Dose** | 50 | 20652.47 | 413.05 | 6.67 | <.001 |
| **Residual** | 216 | 13366.67 | 61.88 |  |  |
| **Total** | 323 | 541034.9 |  |  |  |
| 72 h aphid mortality readings of all aphid clones tested in different lab conditions (Figure S5) at six bacterial concentrations ranging from 10^7^ CFU mL^-1^ to 10^2^ CFU mL^-1^ were considered for General Analysis of variance. The results showed significant variation of mortality between all aphid clones with all test parameters and substantial interactions between all parameters with an exception of bacteria.dose effect between 4106A & UK resistant clones. | | | | | |

**Table S4 Aphid species and their host plants used in this study.**

| **Aphid species** | **Host plant Species** |
| --- | --- |
| *Myzus persicae* | Chinese cabbage (*Brassica napus* L. var *chinensis* cv. Wong Bok) |
| *Aphis fabae* | Pea (*Pisum sativum)* |
| *Aulacorthum solani* | Potato (*Solanum tuberosum*) |
| *Brevicoryne brassicae* | Savoy cabbage (*Brassica oleracea)* |
| *Macrophsiphum albifrons* | Lupin (*Lupinus polyphyllus)* |
| *Nasonovia ribsnigri* | Chinese cabbage (*Brassica napus* L. var *chinensis* cv. Wong Bok) |

**Table S5 *Myzus persicae* clones included in the study and their insecticide resistance mechanisms.**

| **Clone** | **Country** | **Esterases (Est)** | **Modified acetylcholinesterase (MACE)** | **Knock down resistance (Kdr)** | **Super-knock down resistance (Skdr)** | **Amplification of a P450 gene**  **(P450)** | **Mutation of the nicotinic acetylcholine receptor (nAChR mut)** |
| --- | --- | --- | --- | --- | --- | --- | --- |
| 4106A | United Kingdom | SS | SS | SS | SS | SS | SS |
| 4225B | United Kingdom | SS | SS | SS | SS | SS | SS |
| Clone-NS | Germany | SS | SS | SS | SS | SS | SS |
| 794J2 | United Kingdom | RR | SS | RR | SS | SS | SS |
| 5191A | Greece | RR | SR | SS | SS | RR | SS* |
| 5444B | Italy | RR | SS | RR | RR | RR | RR |
| New green | United Kingdom | RR | SR | SR | SR | SS | SS |
| Resistance to insecticide | | Organophosphate and carbamate insecticides | Dimethyl carbamate insecticide | Pyrethroid insecticide | Pyrethroid insecticide | Nicotine and Neonicotinoid insecticide | Neonicotinoid insecticide |
| References | | (Field *et al.,* 1999 | *(*Foster *et al.,* 2000 (Andrews *et al.*, 2004)) | (Martinez-Torres *et al.*, 1997, 1999) | (Eleftherianos, *et al.,* 2008) | (Bass *et al.,* 2011) | (Bass *et al.,* 2013) |

This table is based on an allelic discrimination PCR assay*;* Bass *et al*., 2011*;* Field & Foster, 2002*;* Anstead et al., 2005*,* 2008*.* ***Key****: SS – homozygote susceptible, SR – heterozygote, RR – homozygote-resistant. *Reduced penetration, there is an evidence that this may be a mechanism that confers low levels of resistance to neonicotinoids in this clone.*

**Table S6 Composition of the Mittler aphid artificial diet.**

| **No.** | **Compound** | **mg per 100 mL** |
| --- | --- | --- |
| 1 | Di-Potassium hydrogen orthophosphate | 750 |
| 2 | Magnesium sulphate | 123 |
| 3 | Tyrosine | 40 |
| 4 | L-Asparagine hydrate | 550 |
| 5 | L-Aspartic acid | 140 |
| 6 | L-Tryptophan | 80 |
| 7 | L-Alanine dextro-rotary | 100 |
| 8 | L-Arginine monohydrochloride | 270 |
| 9 | L-Cysteine hydrochloride, hydrate | 40 |
| 10 | L-Glutamic acid | 140 |
| 11 | L-Glutamine | 150 |
| 12 | Glycine | 80 |
| 13 | L-Histidine, free base | 80 |
| 14 | L-Isoleucine (allo free) | 80 |
| 15 | L-Leucine | 80 |
| 16 | L-Lysine -monohydrochloride | 120 |
| 17 | L-Methionine | 40 |
| 18 | L-Phenylalanine | 40 |
| 19 | L-Proline | 80 |
| 20 | L-Serine | 80 |
| 21 | L-Threonine | 140 |
| 22 | L-Valine | 80 |
| 23 | L-Ascorbic acid (Vit. C) | 100 |
| 24 | Aneurine Hydrochloride (Vit. B) | 2.5 |
| 25 | Riboflavin | 0.5 |
| 26 | Nicotinic acid | 10 |
| 27 | Folic acid | 0.5 |
| 28 | (+)-Pantothenic acid (calcium salt) | 5 |
| 29 | Inositol (meso) inactive | 50 |
| 30 | Choline chloride | 50 |
| 31 | Ethylenediaminetetraacetic acid | 1.5 |
| 32 | Fe (III)-Na chelate pure* | 1.5 |
| 33 | EDTA Zn-Na2 chelate pure* | 0.8 |
| 34 | EDTA Mn-Na2 chelate pure* | 0.8 |
| 35 | EDTA Cu-Na2 chelate pure* | 0.4 |
| 36 | Pyridoxine hydrochloride (Vit. B6) | 2.5 |
| 37 | D-Biotin - crystalline | 0.1 |

The solution is made by adding each ingredient one at a time to 100 mL of water with 15 g of

dissolved sucrose, allowing each component to fully dissolve prior to adding the next.

**Figures**


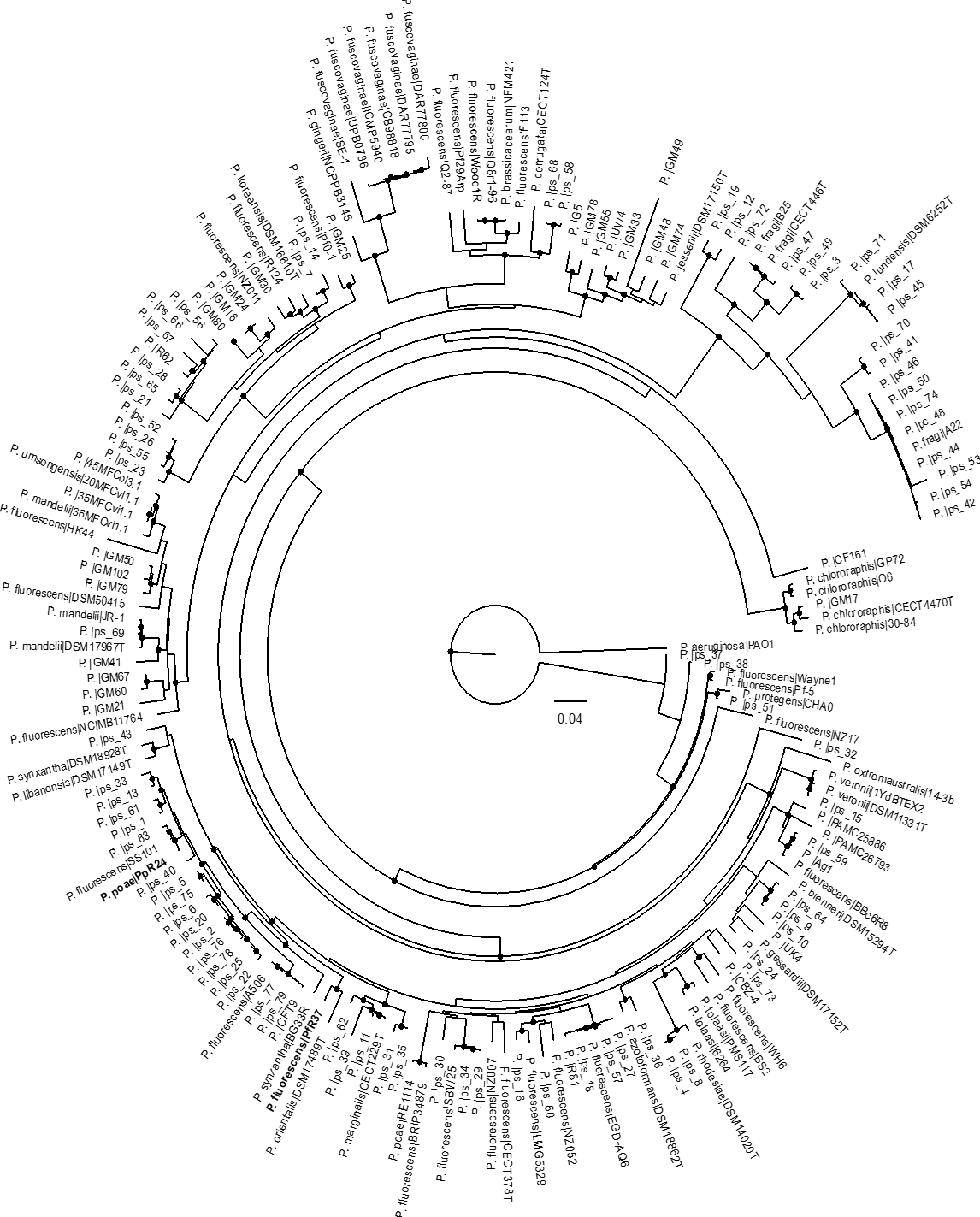

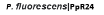


**Figure S1: Maximum Likelihood (ML) tree of 177 *Pseudomonas fluorescens* related strains based on the MLST scheme of Andreani et al. (2014) rooted with *Pseudomonas aeruginosa* strain PAO1**. Trees were drawn to scale and branch length represents the number of base substitutions per site. Nodes annotated with a circle are supported by bootstraps values superior to 70%. The scale bar represents the number of substitutions per site. *P. fluorescens* PpR24 and *P. fluorescens* PfR37 are shown lower left in bold.


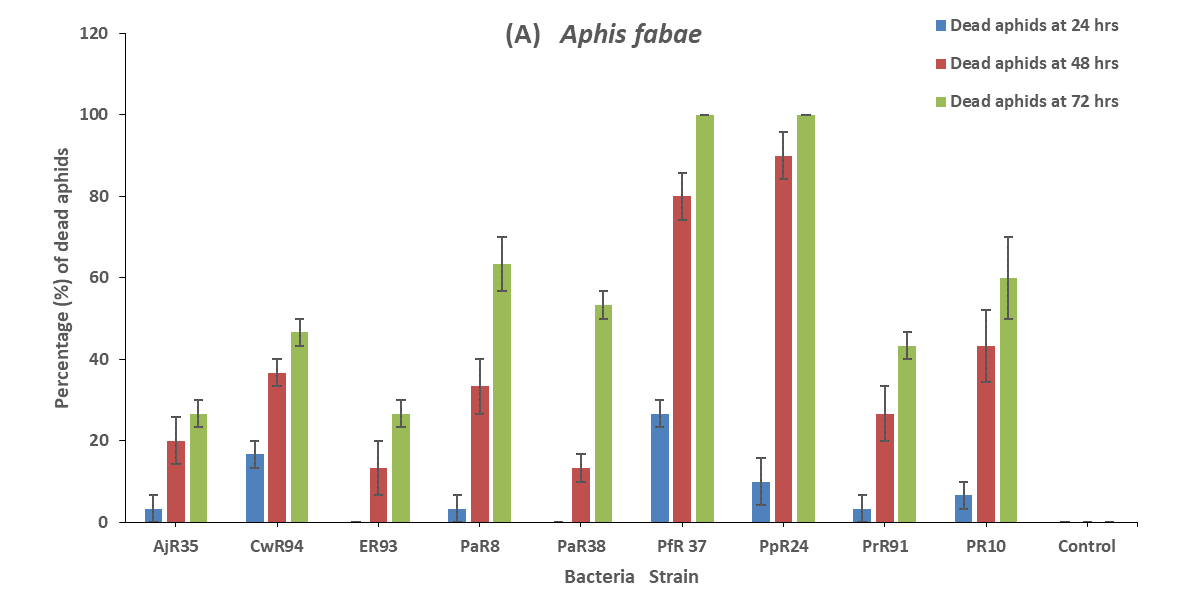

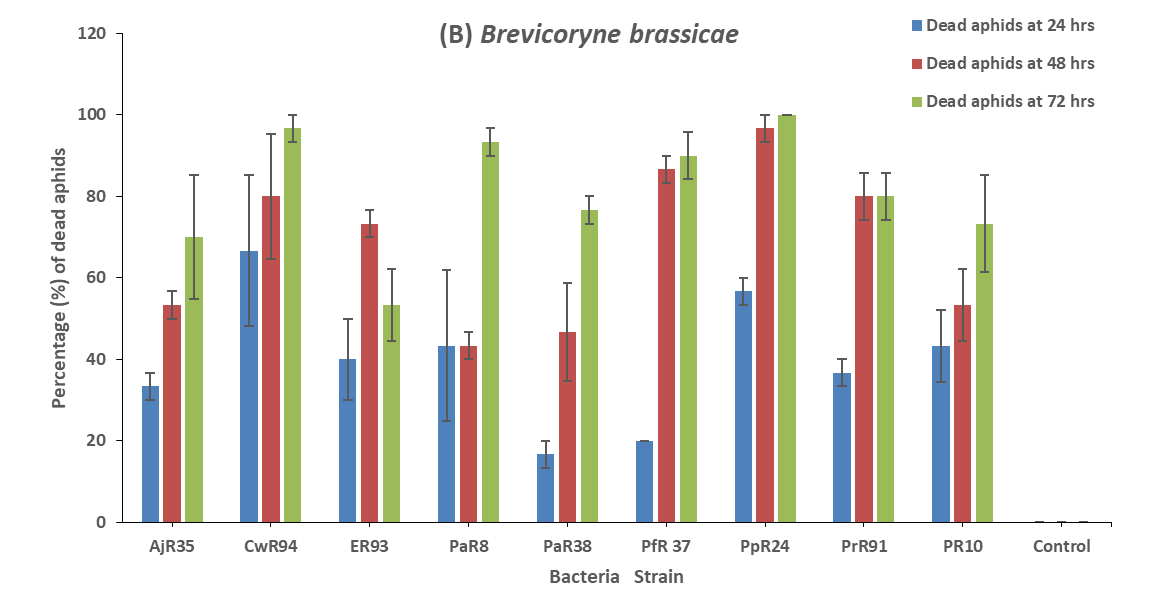

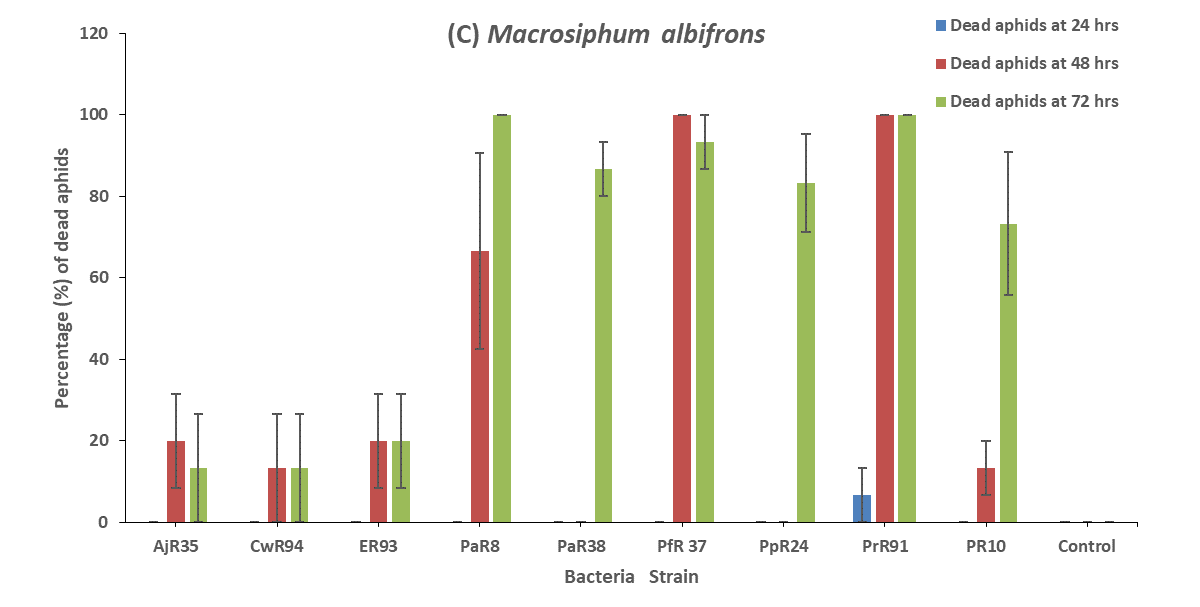


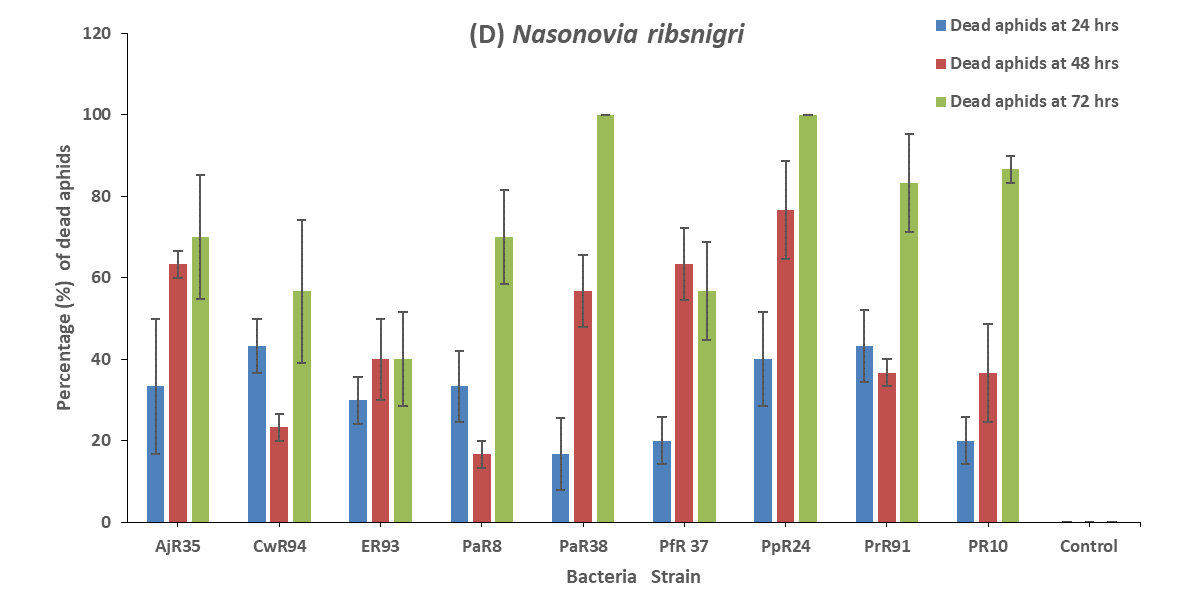

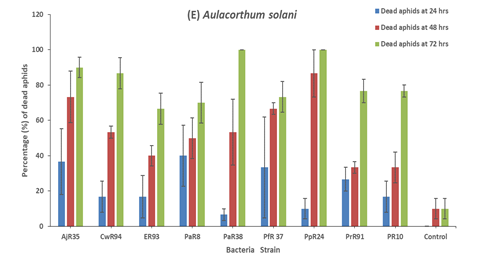


**Figure S2. Differential killing effects of plant-associated bacteria on different aphid species.** Mortality assay showing the percentage of dead aphids (N=10) (A) *Aphis fabae*, (B) *Brevicoryne brassicae*, (C) *Macrosiphum albifrons* (D) *Nasonovia ribsnigri*, (E) *Aulacorthum solani* at 72 hours after ingestion of artificial diet inoculated with various bacterial cells (10^7^ CFU ml^-1^). Error bars represent standard error of the mean of three biological replicates. Bacterial strains tested - *Acinetobacter* sp. AjR35, *Enterobacte*r sp. CwR94, *Enterobacter* sp. ER93, *Pantoea* sp. PaR8, *Pantoea agglomerans* PaR38, *Pseudomonas fluorescens* PfR37, *Pseudomonas fluorescens* PpR24, *Pseudomonas*sp*.* PR10 & *Pseudomonas rhizosphaerae* PrR91. ANOVA detected **statistically significant differences (p<0.05) at 72 hours** and comparison of means by Duncan's multiple comparisons to the control were shown as letters (where different letters on the graphs indicate statistically significant differences) shown in table S2.


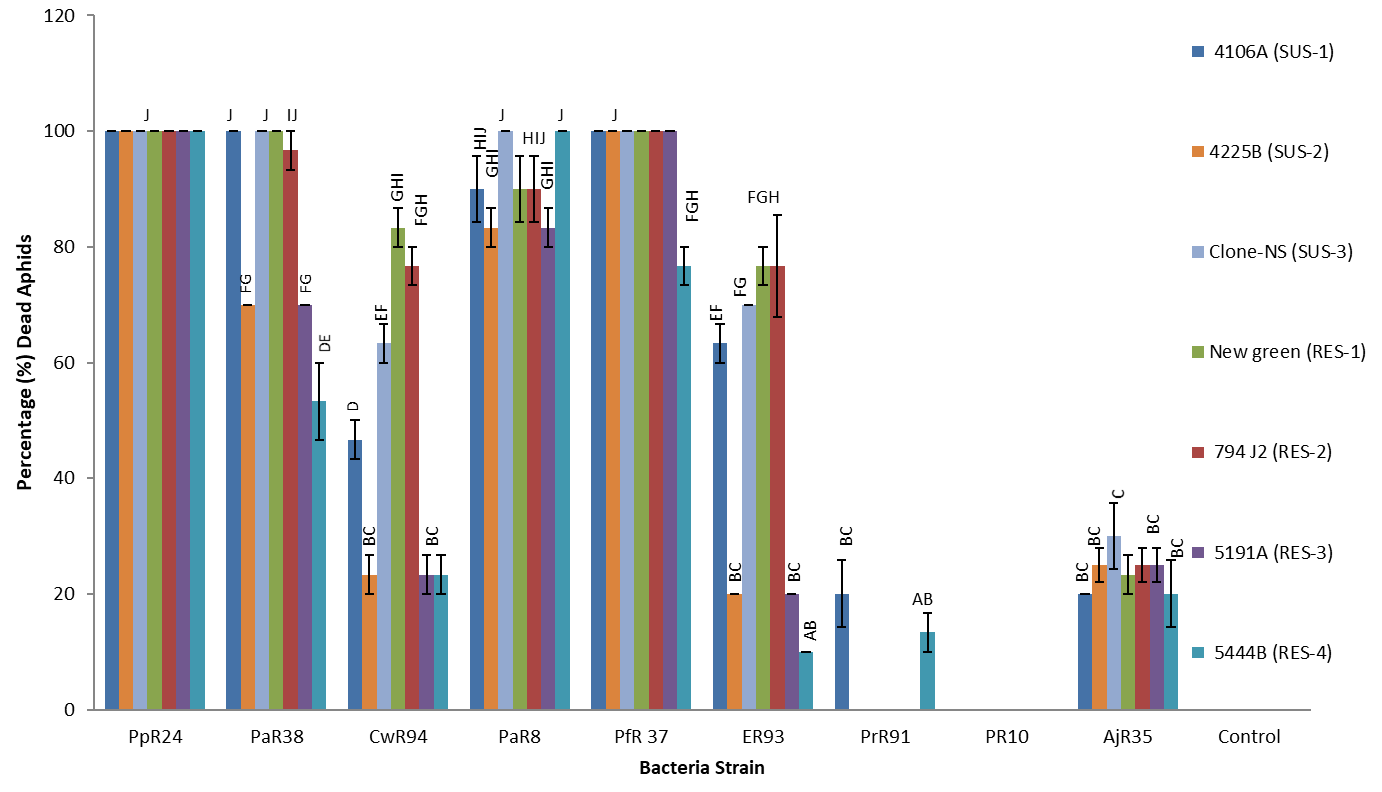


**Figure S3. Assessment of aphid (*Myzus persicae*) mortality by various bacterial species.** Mortality assay showing the percentage of dead aphids (N=10) at 72 h after ingestion of artificial diet inoculated with various bacterial cells (10^7^ CFU ml^-1^). **Control**: Ten aphids were fed in sterile diet with three replicates. Error bars represent standard error of the mean of three biological replicates. ANOVA detected statistically significant differences (p<0.05) and comparison of means by Tukey-Kramer HSD were shown as letters (where different letters on the graphs indicate statistically significant differences). **Aphid clones:** Three susceptible clones “4106A-SUS 1”, “4225B-SUS 2” & “Clone-NS SUS-3” and four resistant clones “New green – RES 1”, “794J2 – RES 2”, ”5191A – RES 3” and “5444B – RES 4”. *Note- Reference clone 4106A 72-hour mortality readings from Figure 1. were used for comparison. **Bacterial strains tested:** *Pseudomonas fluorescens* PpR24, *Pantoea agglomerans* PaR38, *Enterobacte*r sp. CwR94, *Pantoea* sp. PaR8, *Pseudomonas fluorescens* PfR37, *Enterobacter* sp. ER93, *Pseudomonas rhizosphaerae* PrR91, *Pseudomonas*sp*.* PR10 & *Acinetobacter* sp. AjR35.

.


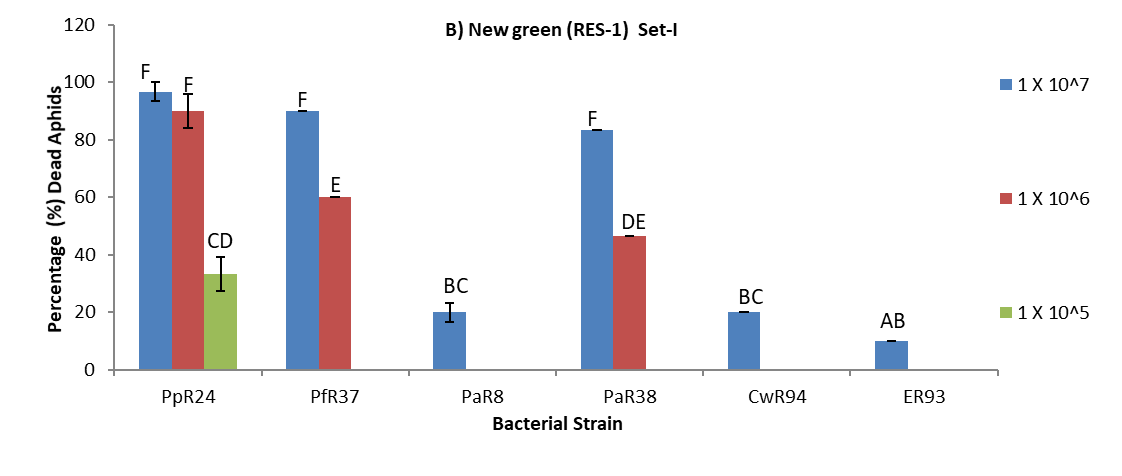

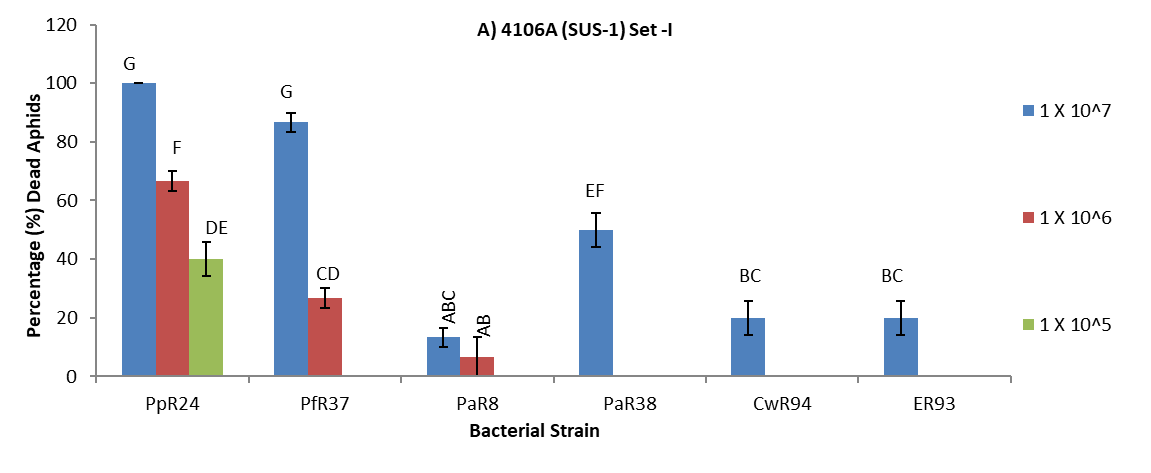


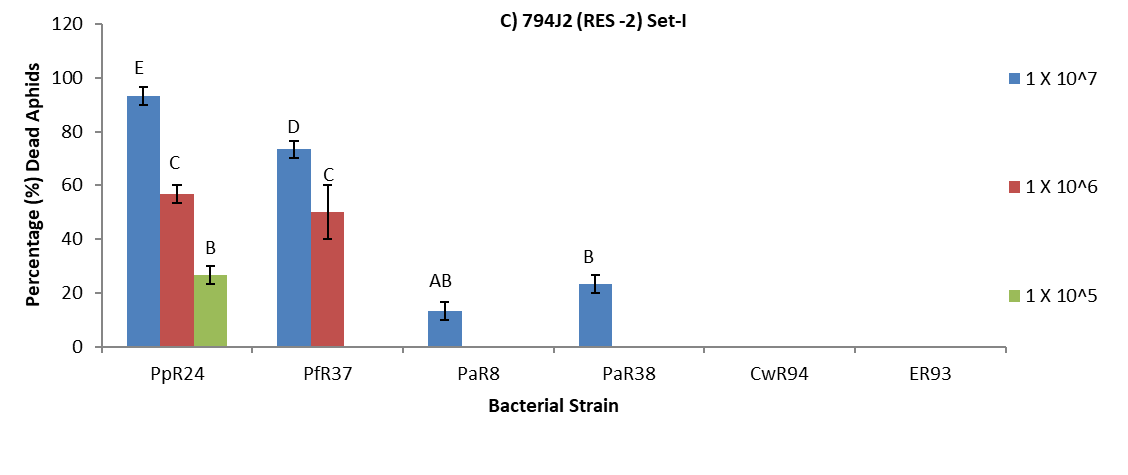


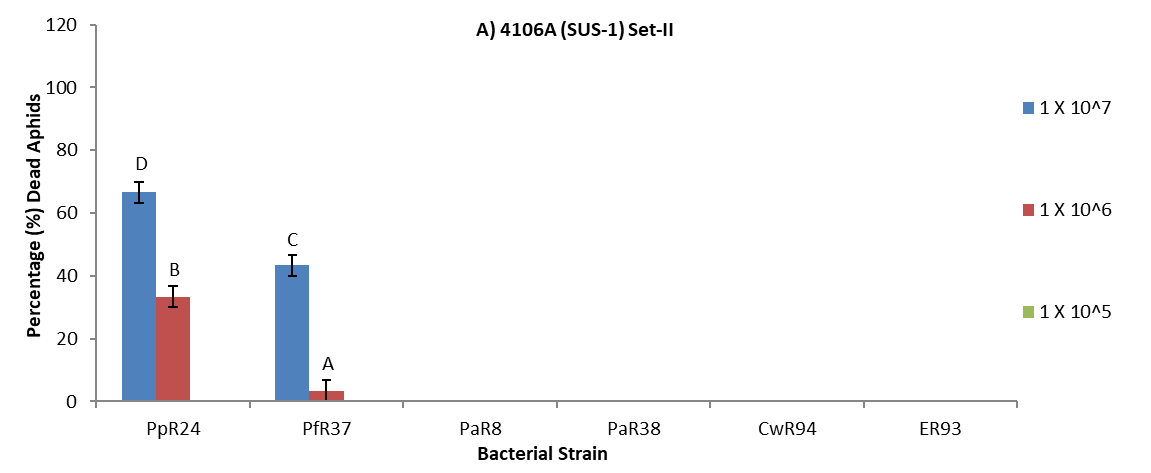

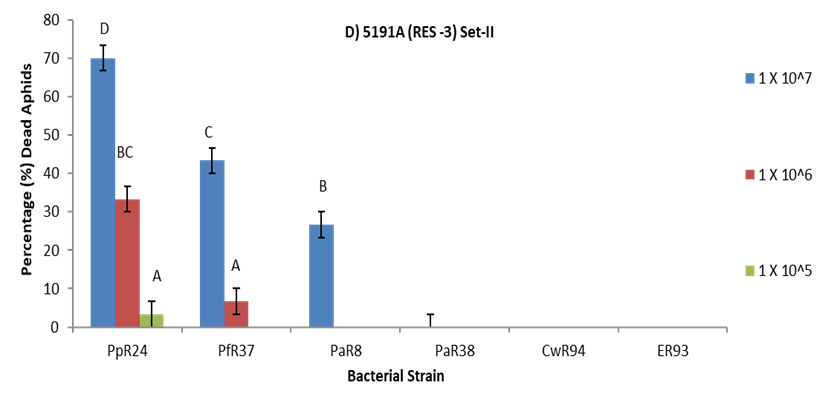


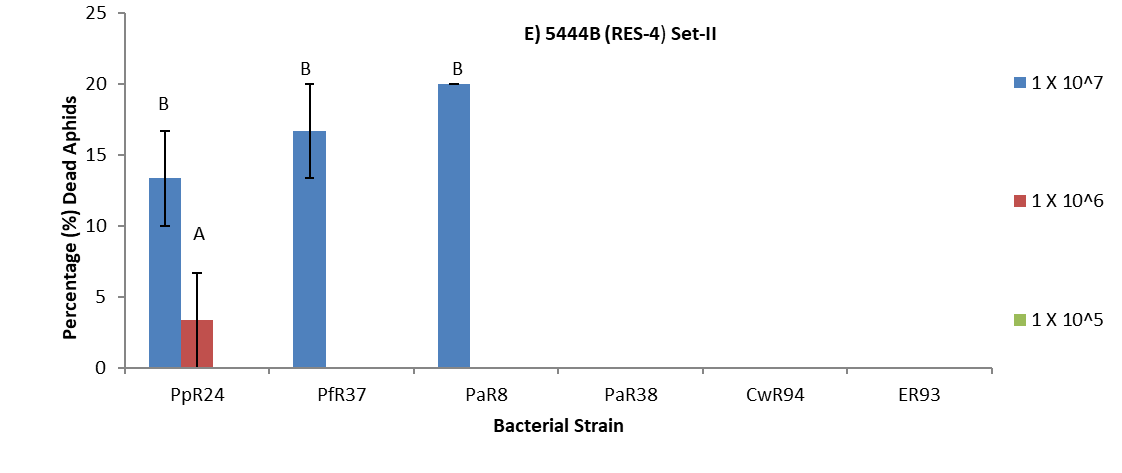


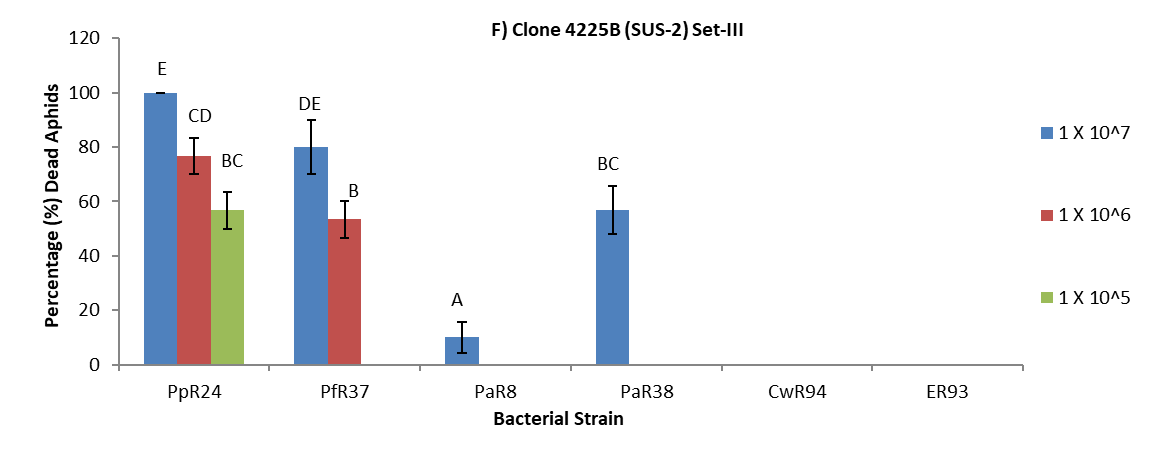

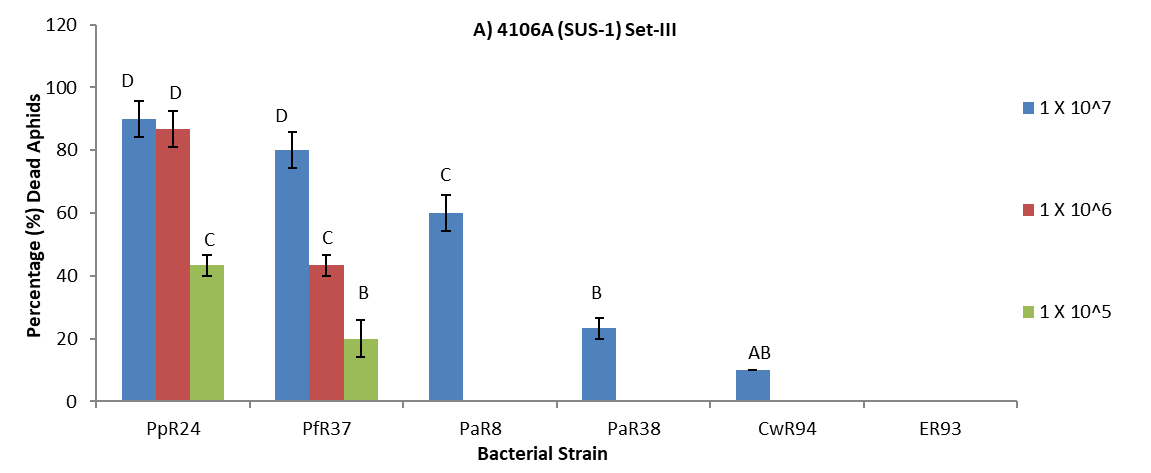


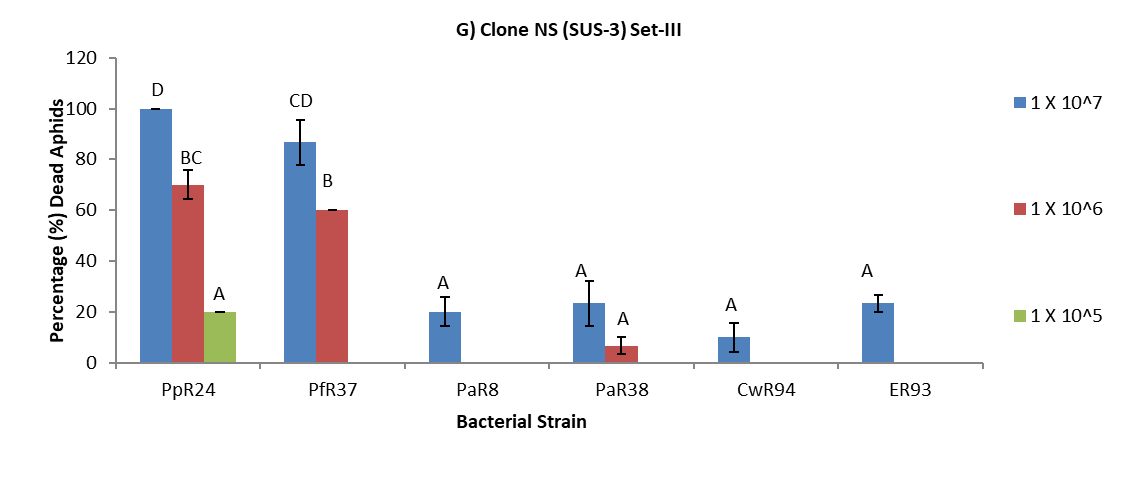


**Figure S4. Effect of bacterial concentration on aphid mortality for various aphid clones after 48 h.**

Three different experiments were carried out based on the availability of growth rooms, with clone 4106A used as a common comparator: Set I Aphid rearing room (University of Reading), Set II Specialist containment Insectary, (Rothamsted Research) and Set III Controlled growth cabinet (University of Reading). Aphid mortality assay showing the percentage (N = 10) of dead aphids {(A) 4106A (SUS-1), (B) New green (RES-1), (C) 794J2 (RES -2), (D) 5191A (RES -3), (E) 5444B (RES-4), (F) Clone 4225B (SUS-2), (G) Clone NS (SUS-3)} after ingestion of artificial diet inoculated with various bacterial species cells at 1 x 10^5^ CFU ml^-1^ (green bars), or 1 x 10^6^ CFU ml^-1^ (red bars), or 1 x 10^7^ CFU ml^-1^ (blue bars), for 48 h. No death was reported in control and lower concentration treated sachets. The data presented are the mean and standard error of three biological replicates. ANOVA detected statistically significant differences (p<0.05) and comparison of means by Tukey-Kramer HSD are shown as letters (different letters on the graphs indicate statistically significant differences). Bacterial strains tested - *Pseudomonas fluorescens* PpR24, *Pseudomonas fluorescens* PfR37, *Pantoea* sp. PaR8, *Pantoea agglomerans* PaR38, *Enterobacte*r sp. CwR94 and *Enterobacter* sp. ER93.


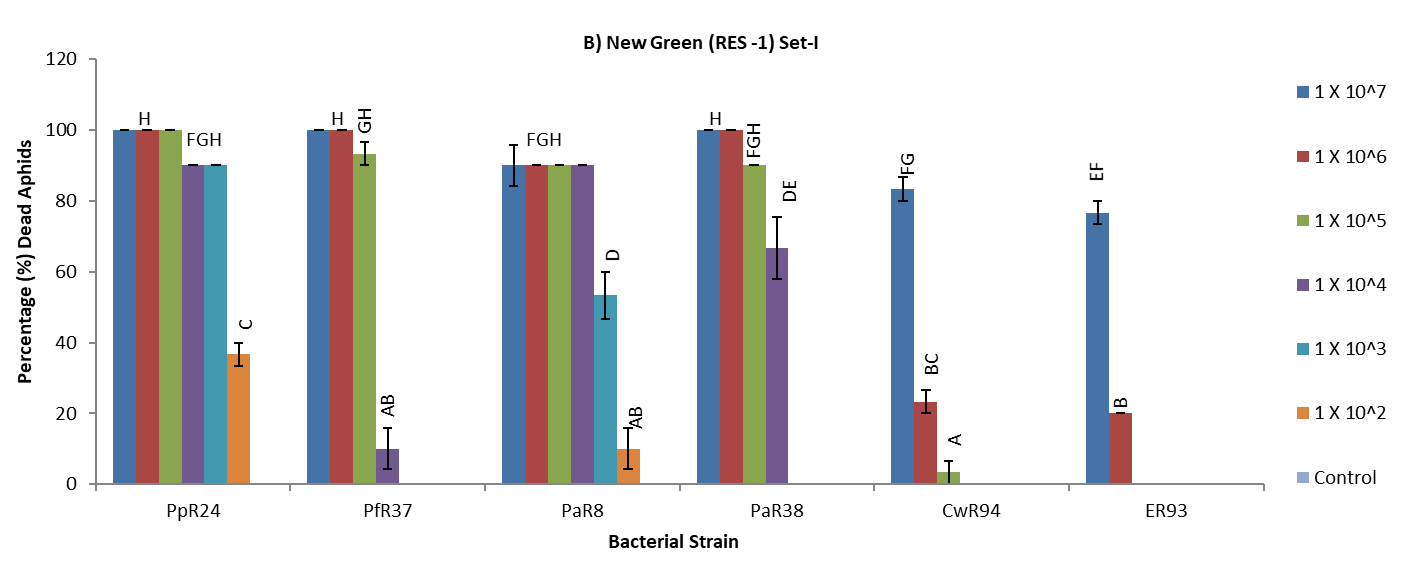

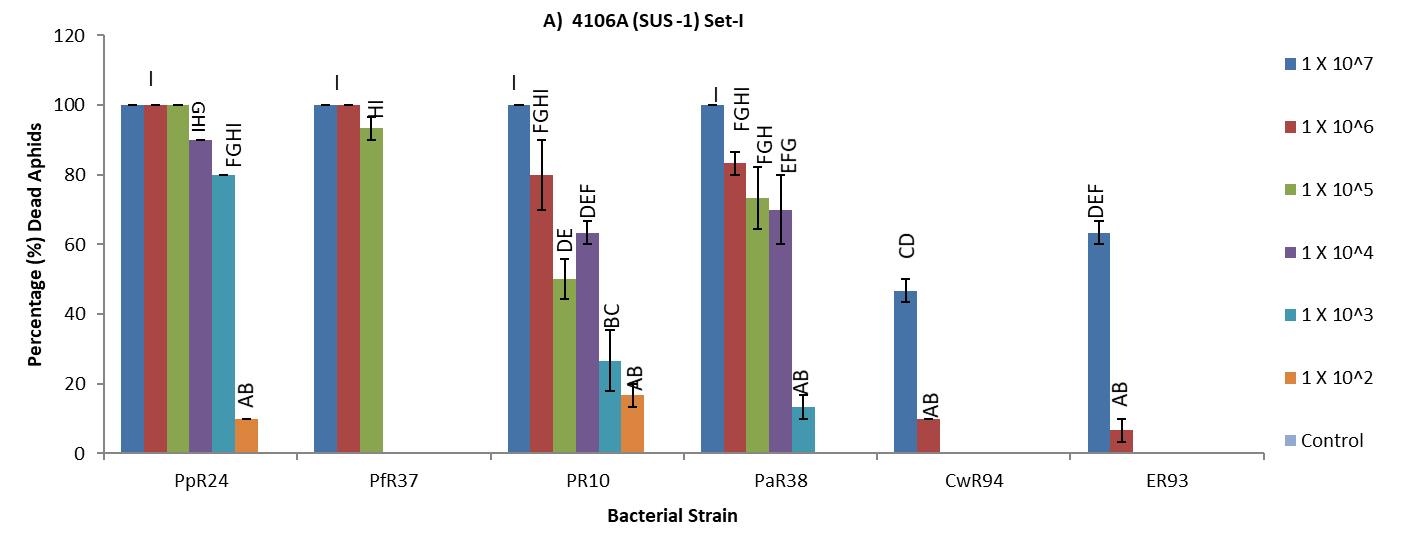


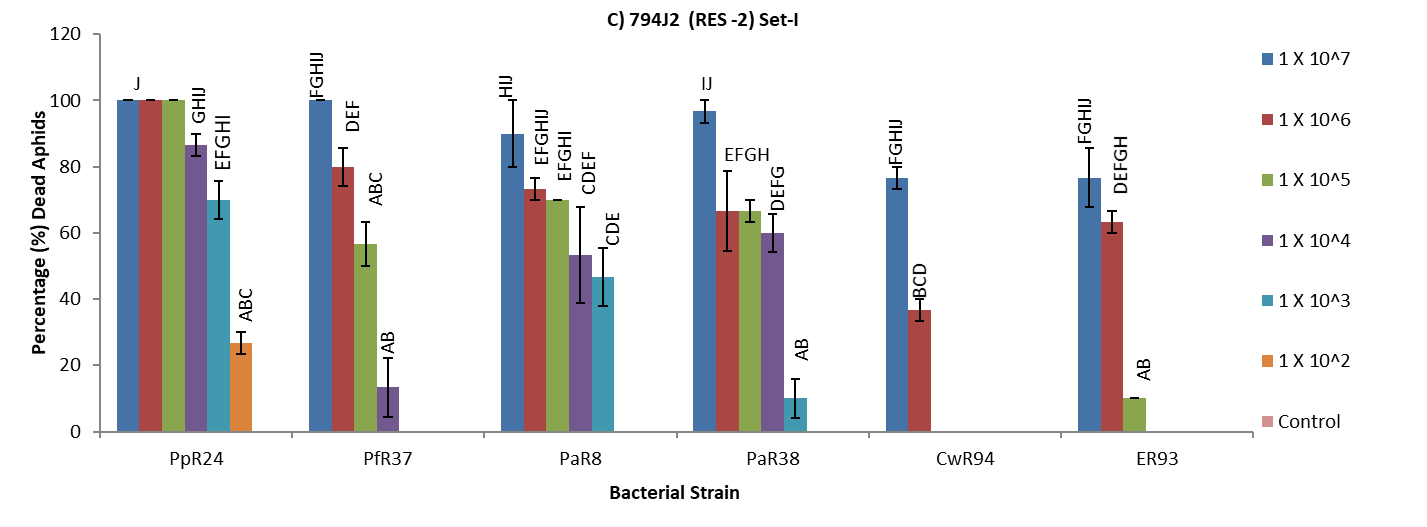


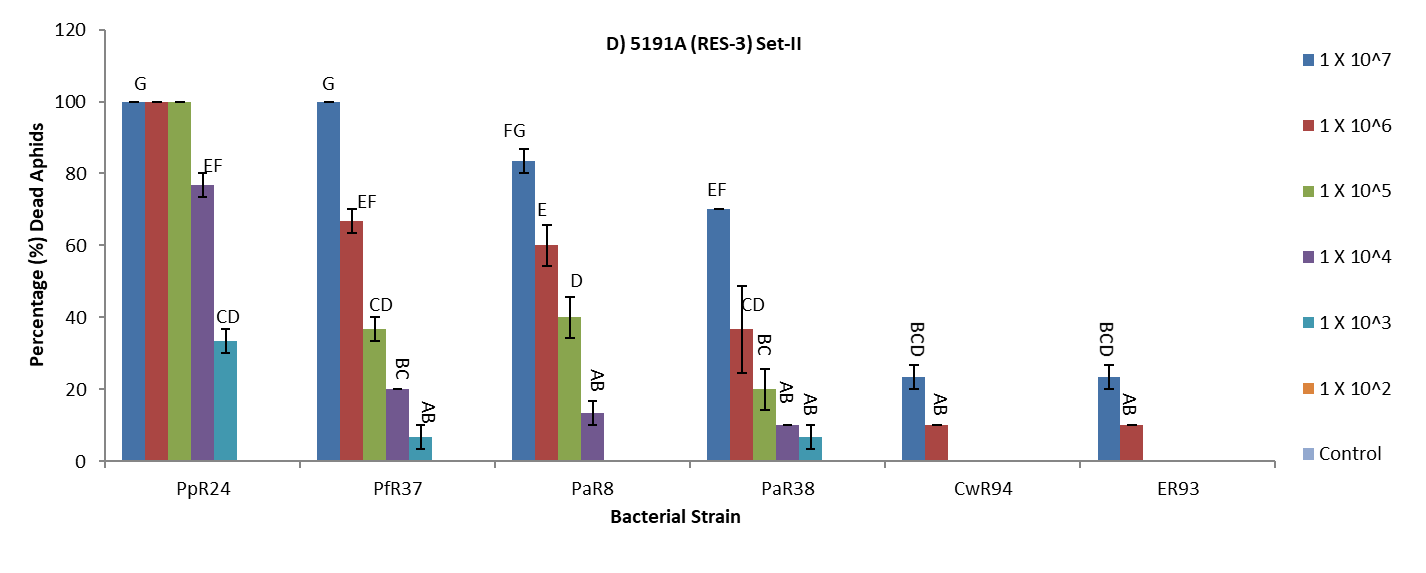

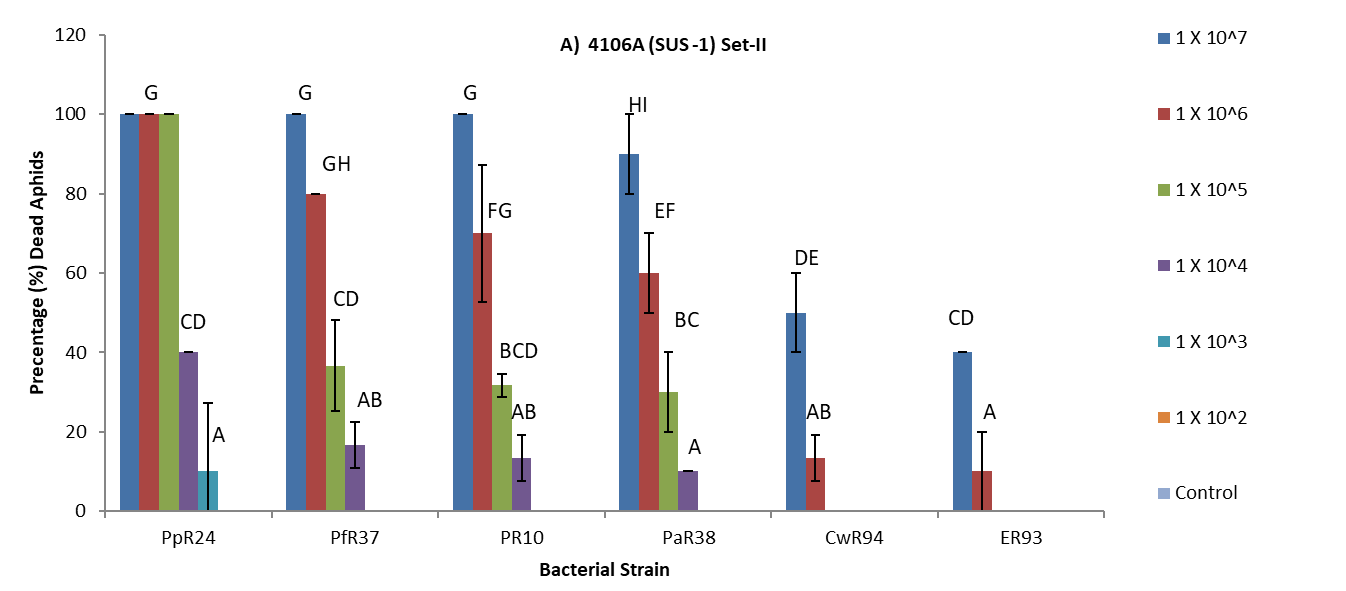


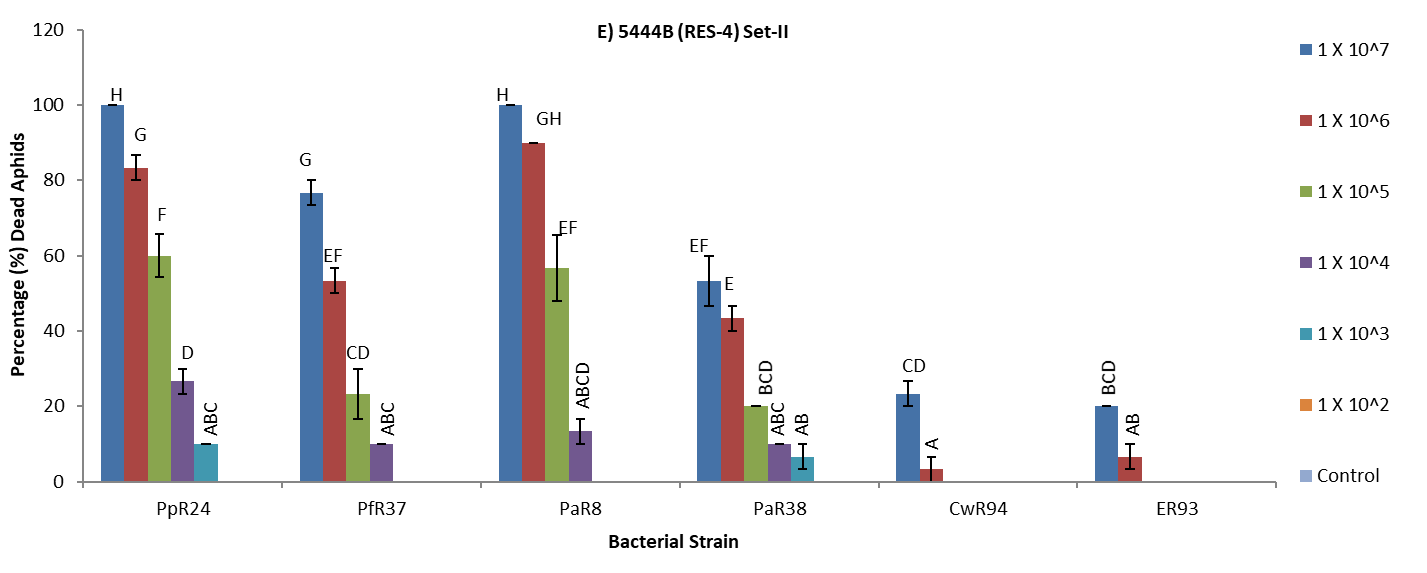


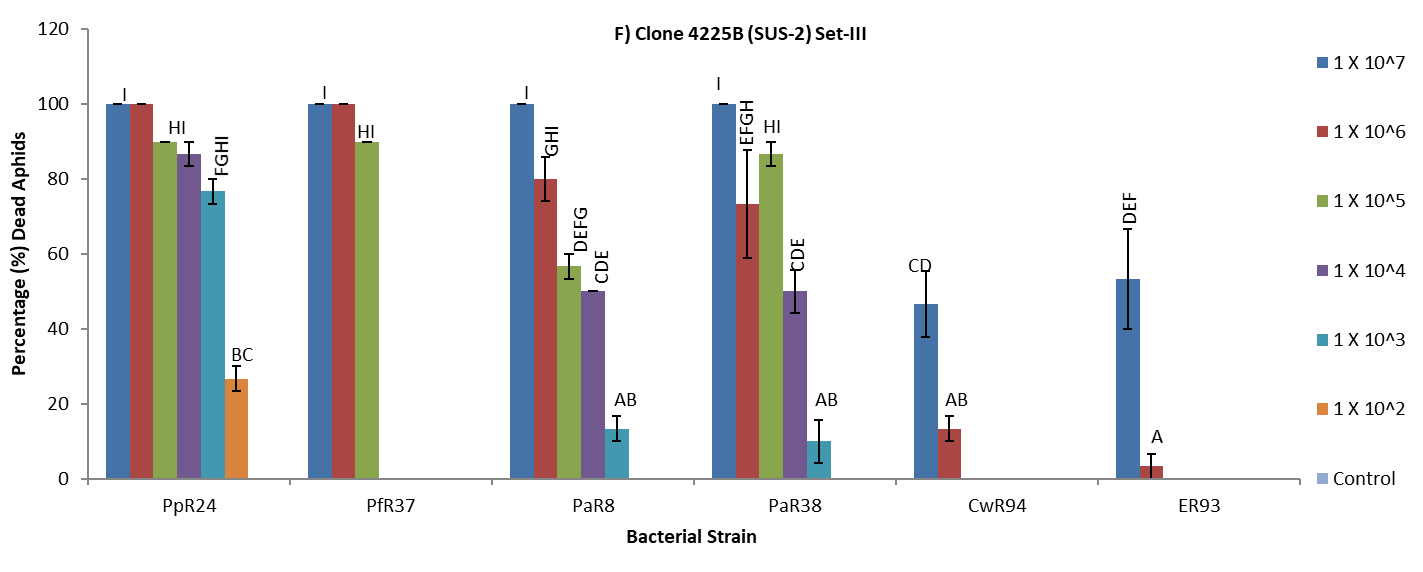

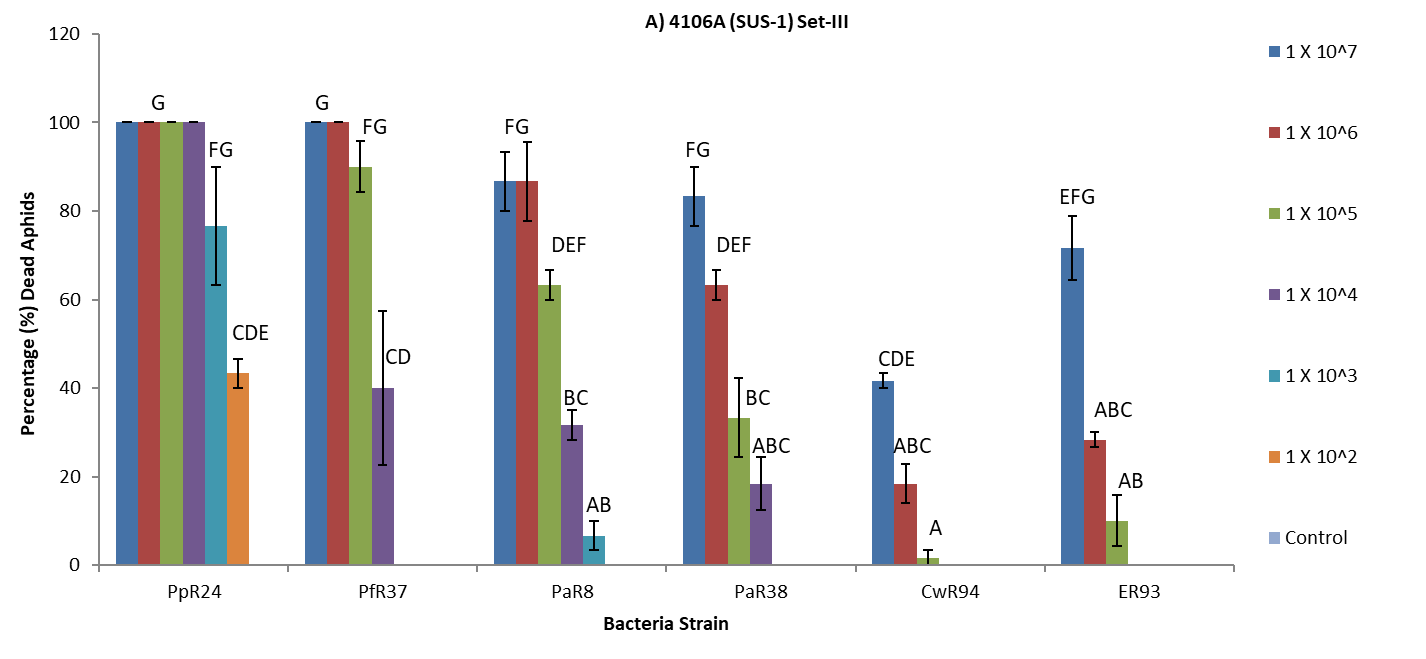


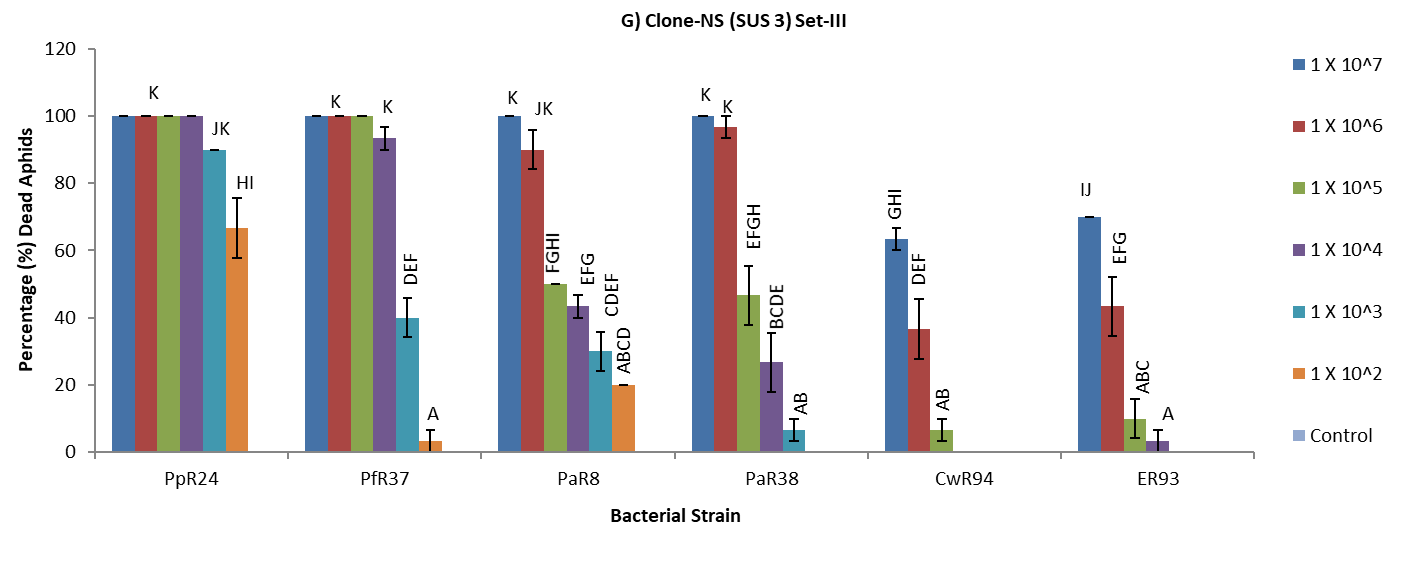


**Figure S5. Effect of bacterial concentration on aphid mortality for various aphid clones after 72 h.**

Three different experiments were carried out based on the availability of growth rooms, with clone 4106A used as a common comparator: Set I Aphid rearing room (University of Reading), Set II Specialist containment Insectary, (Rothamsted Research) and Set III Controlled growth cabinet (University of Reading). Aphid mortality assay showing the percentage (N = 10) of dead aphids {(A) 4106A (SUS-1), (B) New green (RES-1), (C) 794J2 (RES -2), (D) 5191A (RES -3), (E) 5444B (RES-4), (F) Clone 4225B (SUS-2), (G) Clone NS (SUS-3)} after ingestion of artificial diet inoculated with various bacterial species cells at 1 x 10^2^ CFU ml^-1^ (orange bars), 1 x 10^3^ CFU ml^-1^ (light blue bars), 1 x 10^4^ CFU ml^-1^ (purple bars), 1 x 10^5^ CFU ml^-1^ (green bars), or 1 x 10^6^ CFU ml^-1^ (red bars), or 1 x 10^7^ CFU ml^-1^ dark blue bars), for 72 h. No death was observed in control and lower concentration treated sachets. The data presented are the mean and standard error of three biological replicates. ANOVA detected statistically significant differences (p<0.05) and comparison of means by Tukey-Kramer HSD are shown as letters (different letters on the graphs) indicate statistically significant differences. Bacterial strains tested - *Pseudomonas fluorescens* PpR24, *Pseudomonas fluorescens* PfR37, *Pantoea* sp. PaR8, *Pantoea agglomerans* PaR38, *Enterobacte*r sp. CwR94 and *Enterobacter* sp. ER93.


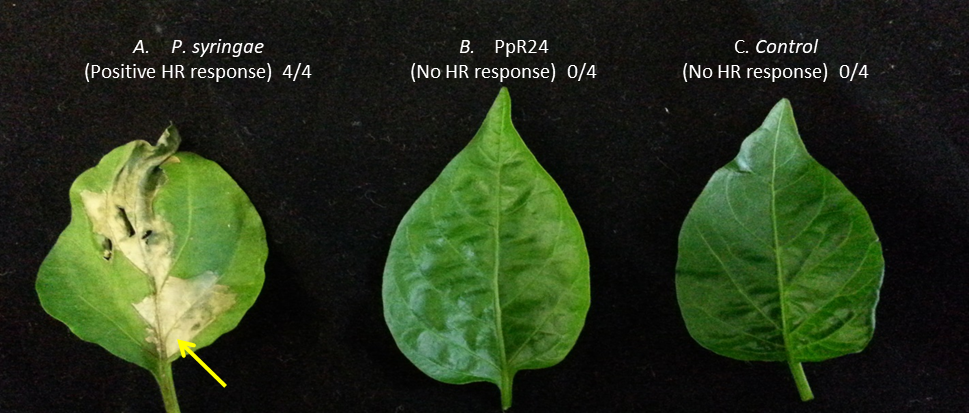


**Figure S6. Assessment of Hypersensitive response (HR) in peppers after foliar spray of different bacteria at 3 day post inoculation (dpi)**. Different bacterial suspensions in water at a concentration of 10^7^ CFU ml^-1^ were sprayed on pepper (*Capsicum annuum* cv. Sapporo (RZ)) plants: A. *P. syringae* pv. *tomato* DC3000 - Positive HR response; B. *P. fluorescens* PpR24 *-* No HR; C. Control (water) No HR. At day 3, the yellow arrow indicates leaf showing HR. The numbers of individual symptomatic plants of the four plants per treatment are indicated.

# **References**

Andrews, M.C., Callaghan, A., Field, L.M., Williamson, M.S., and Moores, G.D. (2004) Identification of mutations conferring insecticide-insensitive AChE in the cotton-melon aphid, *Aphis gossypii* Glover. *Insect Mol Biol* **13**: 555–561.

Anstead, J.A., Williamson, M.S., and Denholm, I. (2005) Evidence for multiple origins of identical insecticide resistance mutations in the aphid *Myzus persicae*. *Insect Biochem Mol Biol* **35**: 249–256.

Anstead, J.A., Williamson, M.S., and Denholm, I. (2008) New methods for the detection of insecticide resistant *Myzus persicae* in the U.K. suction trap network. *Agric For Entomol* **10**: 291–295.

Bass, C., Puinean, A.M., Andrews, M., Cutler, P., Daniels, M., Elias, J., et al. (2011) Mutation of a nicotinic acetylcholine receptor β subunit is associated with resistance to neonicotinoid insecticides in the aphid *Myzus persicae*. *BMC Neurosci* **12**: 51.

Bass, C., Zimmer, C.T., Riveron, J.M., Wilding, C.S., Wondji, C.S., Kaussmann, M., et al. (2013) Gene amplification and microsatellite polymorphism underlie a recent insect host shift. *Proc Natl Acad Sci U S A* **110**: 19460–19465.

Eleftherianos, I., Foster, S.P., Williamson, M.S., and Denholm, I. (2008) Characterization of the M918T sodium channel gene mutation associated with strong resistance to pyrethroid insecticides in the peach-potato aphid, *Myzus persicae* (Sulzer). *Bull Entomol Res* **98**: 183–191.

Field, L.M., Blackman, R.L., Tyler-Smith, C., and Devonshire, A.L. (1999) Relationship between amount of esterase and gene copy number in insecticide-resistant *Myzus persicae* (Sulzer). *Biochem J* **339** : 737–742.

Field, L.M. and Foster, S.P. (2002) Amplified esterase genes and their relationship with other insecticide resistance mechanisms in English field populations of the aphid, *Myzus persicae* (Sulzer). *Pest Manag Sci* **58**: 889–894.

Foster, S.P., Denholm, I., and Devonshire, A.L. (2000) The ups and downs of insecticide resistance in peach-potato aphids (*Myzus persicae*) in the UK. *Crop Prot* **19**: 873–879.

Martinez-Torres, D., Devonshire, A.L., and Williamson, M.S. (1997) Molecular studies of knockdown resistance to pyrethroids: cloning of domain II sodium channel gene sequences from insects. *Pestic Sci* **51**: 265–270.

Martinez-Torres, D., Foster, S.P., Field, L.M., Devonshire, A.L., and Williamson, M.S. (1999) A sodium channel point mutation is associated with resistance to DDT and pyrethroid insecticides in the peach-potato aphid, *Myzus persicae* (Sulzer) (Hemiptera: Aphididae). *Insect Mol Biol* **8**: 339–346.
